# Potential deprescribing indications for antidepressants between 2012 and 2019: repeated cross-sectional analysis in two Scottish health boards

**DOI:** 10.1186/s12916-024-03584-9

**Published:** 2024-09-11

**Authors:** Vita Brisnik, Marietta Rottenkolber, Jochen Vukas, Miriam Schechner, Karoline Lukaschek, Caroline Jung-Sievers, Jochen Gensichen, Ulrich Thiem, Michael Drey, Nils Krüger, Alpana Mair, Bruce Guthrie, Sebastian Fischer, Tobias Dreischulte, for the POKAL Study Group, Peter Falkai, Peter Henningsen, Markus Bühner, Helmut Krcmar, Gabriele Pitschel-Walz, Antonius Schneider, Kirsten Lochbuhler, Barbara Prommegger, Andrea Schmitt, Katharina Biersack, Constantin Brand, Christopher Ebert, Julia Eder, Feyza Gökce, Carolin Haas, Lisa Pfeiffer, Lukas Kaupe, Jonas Raub, Philipp Reindl-Spanner, Hannah Schillok, Petra Schönweger, Clara Teusen, Marie Vogel, Victoria von Schrottenberg, Puya Younesi

**Affiliations:** 1 Institute of General Practice and Family Medicine, LMU University Hospital, LMU Munich, Munich, Germany; 2 Graduate Program “POKAL - Predictors and Outcomes in Primary Care Depression Care” (DFG-GrK2621), Munich, Germany; 3 Institute of Medical Data Processing, Biometrics and Epidemiology (IBE), Faculty of Medicine, LMU Munich, Munich, Germany; 4 Pettenkofer School of Public Health, LMU Munich, Munich, Germany; 5 Department of Geriatrics, Albertinen-Haus, Hamburg, Germany; 6 Department of Medicine IV, Geriatrics, LMU University Hospital, LMU Munich, Munich, Germany; 7 Department of Cardiology, German Heart Center Munich, Technical University Munich, Munich, Germany; 8 Deutsches Zentrum Für Herz- Und Kreislaufforschung (DZHK), Partner Site Munich Heart Alliance, Munich, Germany; 9 Effective Prescribing and Therapeutics Division, Scottish Government, Edinburgh, Scotland, UK; 10 Advanced Care Research Centre, Usher Institute, The University of Edinburgh, Edinburgh, UK; 11 Psychiatric Services Lucerne, Lucerne, Switzerland

**Keywords:** Antidepressants, Deprescribing, Long-term use, Adverse drug events

## Abstract

**Background:**

Antidepressants have a pivotal role in the treatment of many psychiatric disorders, but there are concerns about long-term use and adverse effects. The objectives of this study were (1) to examine time trends in antidepressant use, (2) to estimate the prevalence of long-term and potential high-risk antidepressant use, and (3) to examine patient characteristics associated with potential deprescribing indications (PDIs) (i.e., simultaneous long-term and potential high-risk antidepressant use).

**Methods:**

Repeated population-based cross-sectional study for all 609,299 people aged ≥ 18 years resident in the Tayside or Fife regions of Scotland. The prevalence of antidepressant use was examined on June 30th (index date) of each year from 2012 to 2019, while the prevalence of long-term and potential high-risk use as well as PDIs was assessed and compared on the same dates in 2012 and 2019. Binary logistic regression modeling was used to examine patient characteristics associated with PDIs.

**Results:**

Antidepressant use increased by 27% from 12.0 to 15.3% among adult residents between 2012 and 2019. While the proportion of antidepressants users dispensed ≥ 1 antidepressant for > 2 years increased from 54.3 to 61.9% between 2012 and 2019, the proportion of antidepressant users triggering ≥ 1 indicator of potential high-risk use decreased slightly from 37.9 to 34.7%. In 2019, potential high-risk use most commonly related to indicators targeting fall risk (16.0%), cardiovascular risks (14.1%), insomnia (10.6%), and risk of orthostatic hypotension (8.6%). More than 1 in 4 (25.8%) antidepressant users had PDIs. The main risk factors associated with PDIs included increasing age (65–79, adjusted OR 14.12; 95% CI, 13.15–15.17), increasing number of drugs taken concomitantly (≥ 15 drugs, adjusted OR 7.37; 95% CI, 6.71–8.10), use of tricyclic antidepressants (≥ 50 mg) (adjusted OR 5.49; 95% CI, 5.02–6.01), and concomitant use of ≥ 2 antidepressants (adjusted OR 5.52; 95% CI, 5.20–5.85).

**Conclusions:**

Long-term and potential high-risk use of antidepressants is widespread, and potential deprescribing indications (PDIs) are increasing, suggesting the need for a critical review of their ongoing use by clinicians. If deemed necessary, future deprescribing interventions may use the criteria applied here for identification of patients with PDIs and for evaluating intervention effectiveness.

**Supplementary Information:**

The online version contains supplementary material available at 10.1186/s12916-024-03584-9.

## Background

Antidepressants are among the most commonly prescribed prescription drugs globally and have a pivotal role in the treatment of many psychiatric disorders, particularly in moderate to severe symptoms of depression and anxiety disorders [1–4]. For relapse prevention, clinical guidelines recommend treatment up to two years (or more depending on the number of recurrent episodes) [3]. However, longer than recommended use of antidepressants is prevalent [5–9] and has been identified as a key driver for the global increase in antidepressant use [10, 11]. For example, studies in Switzerland, the Netherlands, and UK have found rates of long-term use of more than 40% [6, 7, 9], while the median duration has been reported to exceed 2 years in the UK [9] and 5 years in the USA [12]. This raises safety concerns, particularly in patients at increased risk of adverse drug reactions, such as older people with polypharmacy [13–15]. Several studies also suggest that a substantial proportion of antidepressant users in primary care may be using these drugs without a significant benefit, including those with mild depression [16–19], where antidepressant use is discouraged by guidelines [1].

The concept of deprescribing denotes a systematic approach to reducing, discontinuing, or switching medication [20] for those who no longer need a medicine, do not benefit from it, or may be at increased risk of adverse effects. The process of deprescribing antidepressants can be complex and time consuming, as it may require a nuanced balancing of benefits and risks of continued antidepressant use versus cessation, with the latter including consideration of potential risk of disease recurrence and withdrawal symptoms [21]. In addition, a barrier to prescribers implementing deprescribing is lack of guidance on when it is appropriate to consider it, especially when patients are at increased risk of serious adverse effects, e.g., acute bleeding or fall injuries, but have not experienced them [22, 23].

To support prescribers in reviewing the use of antidepressants, a set of explicit criteria of potentially inappropriate antidepressant use (indicators) was recently developed in an expert consensus process [24], covering clinical situations of potential high-risk and overprescribing. Overprescribing criteria identify patients who use antidepressants for indications where they have little benefit [18] or for longer durations than recommended [3], while potential high-risk prescribing criteria identify patients at increased risk of adverse drug reactions, such as falls, gastrointestinal bleeding, cardiovascular adverse effects, and hyponatremia [25–29].

The objectives of this study were (1) to examine time trends in antidepressant use and to use a recently developed consensus criteria-set, (2) to estimate the prevalence of long-term and potential high-risk antidepressant use, and (3) to examine patient characteristics associated with potential deprescribing indications (PDIs) (i.e., simultaneous long-term and potential high-risk antidepressant use).

## Methods

### Study design

We conducted a repeated population-based cross-sectional study of community-dispensed antidepressant prescribing for all 609,299 people aged 18 years or older resident in the Tayside and Fife regions of Scotland. In order to examine time trends in antidepressant use, we estimated exposure on a given index date of each year from 2012 to 2019, and chose June 30th as the mid-year time point. In order to estimate the prevalences of long-term and potential high-risk use (separate and simultaneous) on the same dates in 2012 and 2019, we used indicators previously developed in an expert consensus process [24] and compared these rates in 2012 and 2019. We used a binary logistic regression modeling to examine patient characteristics associated with simultaneous long-term and potential high-risk use among antidepressant users in 2019.

### Data source

Data were obtained from a large, population-based data set from Scotland provided by the University of Dundee/National Health Service (NHS) Tayside Health Informatics Centre. The data set included prescriptions by general practitioners (GPs) dispensed by community pharmacies (drug names and British National Formulary (BNF) codes [30]) and demographic data (date of birth, gender, registration and de-registration date with NHS Tayside or NHS Fife, date of death, socioeconomic status (according to the Scottish Index of Multiple Deprivation) [31], area of patient’s residence (classified by the Scottish Executive Urban–rural Classification) [32]), as well as hospital admissions (including ICD-10 coded diagnoses) for all people aged ≥ 18 years residing in the Tayside and Fife regions of Scotland. Tayside and Fife have a total population of approximately 900,000 people and are broadly representative of Scotland in terms of age and socioeconomic status. In order to receive public health care, each resident is registered with a single NHS general practice, who is responsible for all community prescribing to patients. Individual study ethical review was not required as all analyses were conducted using non-identifiable data and were carried out in the ISO27001 and Scottish Government approved Health Informatics Centre (HIC) Safe Haven (www.hic.dundee.ac.uk) whose standard operating procedures have been approved by the Caldicott Guardian on behalf of the NHS data controllers.

### Definitions

#### Antidepressant use

We classified antidepressants as tricyclic antidepressants (TCA), selective serotonin reuptake inhibitors (SSRI), selective serotonin-norepinephrine reuptake inhibitors (SNRI), noradrenergic and specific serotonergic antidepressants (NASSA), monoamine oxidase inhibitors (MAOIs), and other ADs (trazodone, agomelatine, nefazodone, reboxetine, vortioxetine, bupropion). In order to determine exposure on the index date, we considered any dispensations of antidepressants in the 2nd quarter (i.e., three months from April 1st to June 30th) on the basis that usual dispensing intervals in the UK are 8 weeks and there may be irregularities, e.g., due to holidays.

#### Long-term use

The indicator set developed by a previous expert-based consensus process [24] originally included 25 indicators of long-term use for indications of depression, anxiety, and insomnia as well as otherwise potentially unnecessary use of antidepressants, such as for indications without evidence of relevant benefit (e.g., mild depression) or at higher doses than indicated (e.g., ≥ 50 mg TCA for insomnia). The definitions of antidepressant long-term use vary depending on indication (i.e., from > 8 weeks for treatment of insomnia to > 2 years for treatment of recurrent depression). However, the data source used did not contain information on indications for treatment. Long-term use was therefore conservatively defined as a single measure of continuous prescription for > 2 years, i.e., for 8 quarters or more prior to index dates in 2012 and 2019 (while allowing for a grace period of up to one quarter). Details are provided in the Additional file 1: Fig. S1.

#### Potential high-risk use

The indicator set originally included 37 indicators of potential high-risk antidepressant use [24], with each indicator identifying patient risk factors (i.e., advanced age, comedication, daily dose [in case of tricyclic antidepressants only], and/or comorbidity) that may increase the risk of antidepressant adverse drug reactions. To identify relevant comorbidities in the absence of ambulatory care diagnoses in the data source, we either used hospital diagnoses (e.g., hospital admission with gastrointestinal ulcer or bleeding, falls or fall injuries) or drug proxies (e.g., previous use of antidementia drugs as a proxy for dementia). However, there were no reliable drug proxies for 9 indicators (e.g., tachycardia, dizziness, hepatic impairment, or angle closure glaucoma), which were therefore omitted from this analysis. The complete list of the 28 operationalized indicator definitions (including ICD-10 codes for hospital diagnoses and BNF Codes for medication) is provided in the Additional file 2: Table S1, S2, and S3.

#### Potential deprescribing indications (PDIs)

Although any long-term use or potential high-risk prescribing of antidepressants may justify a critical review of antidepressant use, we opted to define PDIs more conservatively as instances where patients were identified to be simultaneously exposed to both long-term and potential high-risk use.

### Statistical methods

To determine time trends in antidepressant use, we included individuals who were aged 18 years or older and registered with a GP in the Tayside or Fife regions at any point during the three months prior to index dates (i.e., April 1st to June 30th) of each year from 2012 through 2019 (denominator). We calculated the proportion of all adults who had been exposed to antidepressants on June 30th in each year. The prevalence was calculated per 100 people and for each antidepressant group separately. The prevalence of antidepressant users was stratified by gender, age group (18–39, 40–64, 65–79, 80–100), type of antidepressant drug class (SSRI, TCA, SNRI, NASSA, MAOI, other ADs), and socioeconomic status (1 = most deprived, 5 = least deprived, according to the Scottish Index of Multiple Deprivation) as well as residence (large urban area, urban area, accessible rural area, and remote rural area) according to the Scottish Executive Urban–rural Classification. The relative risks between 2019 vs 2012 (and 95% confidence intervals (CI)) were calculated as non-standardized (crude) and age-sex standardized percentage rates to account for changes in population demographics between 2012 and 2019 (2019 data directly age-sex standardized to 2012 population structure).

To determine the prevalence of long-term and potential high-risk use, separately and simultaneous (PDIs), we considered the proportion of all antidepressant users on each index dates in 2012 and 2019, who triggered one or more of the above. Among prevalent antidepressant users, the absolute numbers and rates of patients triggering long-term use, potential high-risk use or PDIs were stratified by gender, age group, type of antidepressant drug class, and socioeconomic status as well as residence and rates compared between 2012 and 2019. The relative risk between 2019 vs 2012 (and 95% confidence intervals (CI)) were calculated as non-standardized (crude) and age-sex standardized percentage rates.

For 2019, the associations between patient characteristics and having PDIs were examined using binary logistic regression models. Initially, unadjusted odds ratios (ORs) with 95% CIs were calculated with subsequent multivariate analysis. Patient variables considered were age group, gender, total number of medication groups dispensed in the index quarter of 2019 (1–4, 5–9, 10–14, 15 + ; defined as subsections of the BNF, typically containing a single class of agent with similar mechanism of action as described by reference [33]), type of antidepressant regimen as defined by the indicators (SSRI, SNRI, TCA (prescribed ≥ 50 mg), mirtazapine (prescribed ≤ 15 mg—low dose), NASSA (mirtazapine > 15 mg, mianserin, maprotiline), or other antidepressants in monotherapy or a combined use of ≥ 2 antidepressants)), socioeconomic status, and residence. Data management and statistical analyses were performed using SPSS (version 25, IMB Corporation 2018). A *p*-value < 0.05 was considered statistically significant.

### Sensitivity analysis

We conducted four sensitivity analyses (SAs) to test the robustness of our findings. In SA1, we restricted the definition of long-term use to the use of antidepressants in each of 8 consecutive quarters (i.e., without grace periods) to examine potential overestimation of long-term use (by allowing grace periods of one quarter). We also explored the impact of more conservative definitions of high-risk use co-prescriptions with high prevalence (i.e., co-prescription of antidepressants with two or more rather than one or more fall risk increasing drug in SA2 and co-prescription of certain antidepressants with two or more rather than one or more drug known to increase the risk of torsades des pointes in SA3). For SA4, we restricted the definition of high-risk use to indicators which had achieved the highest consensus ratings (median of 8 or 9 on a 9-point Likert scale) within the expert panel [24], in order to examine potential overestimation of high-risk use.

## Results

### Study population

There were 614,421 individuals aged ≥ 18 years resident and registered in the Tayside and Fife regions in the 2nd quarter of 2012, with a mean (standard deviation (SD)) age of 50.3 (18.7) years, decreasing to 607,215 in 2nd quarter of 2019, with a mean (SD) age of 51.5 (19.0) years (Table 1). The proportion of residents aged ≥ 65 years rose from 25.0% in 2012 to 27.6% in 2019.

**Table 1:** Characteristics of the study population

	Second quarter 2012	Second quarter 2019
	No. of patients (crude %)	
Total	614,421	607,215
Sex		
Women	315,046 (51.3)	310,363 (51.1)
Men	299,375 (48.7)	296,852 (48.9)
Mean age (SD)	50.3 (18.7)	51.5 (19.0)
Age groups (years)		
18–39	191,155 (31.1)	186,719 (30.8)
40–64	269,912 (43.9)	252,871 (41.6)
65–79	111,195 (18.1)	121,229 (20.0)
80–100	42,159 (6.9)	46,396 (7.6)
Deprivation quintile^a^		
1 (most deprived)	95,057 (15.5)	95,467 (15.7)
2	106,030 (17.3)	104,903 (17.3)
3	114,450 (18.6)	112,059 (18.5)
4	154,800 (25.2)	150,633 (24.8)
5 (least deprived)	110,532 (18.0)	107,279 (17.7)
Residence^a,b^		
Large urban area	118,366 (19.3)	115,689 (19.1)
Urban area	258,032 (42.0)	253,421 (41.7)
Accessible rural area	179,545 (29.2)	177,669 (29.3)
Remote rural area	24,926 (4.1)	23,562 (3.9)

^a^Deprivation and residence missing for 33,552 (5.5%) people registered in the index quarter of 2012 and 36,874 (6.1%) in 2019

^b^Scottish Executive Urban–Rural Classification

### Changes in the prevalence of antidepressant use between 2012 and 2019

**Fig. 1:**
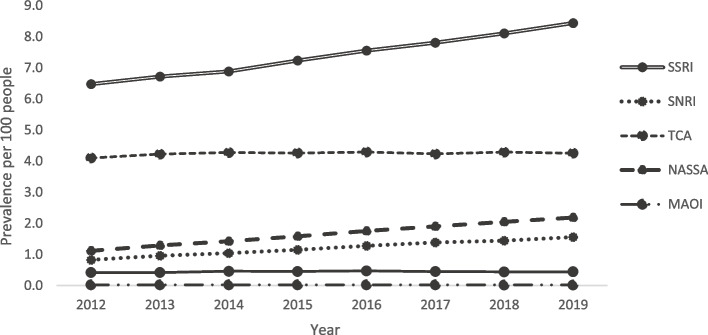
Proportion of residents aged ≥ 18 years dispensed ≥ 1 antidepressant between 2012 and 2019

Between 2012 and 2019, the crude proportion of adults dispensed one or more antidepressants increased from 12.0 to 15.3% with an age-sex standardized relative risk (sRR) of 1.27 [95% CI, 1.26–1.28]. Figure 1 shows the proportion of adults, who were dispensed one or more antidepressant drug class from 2012 to 2019. There were marked increases in SSRI, SNRI, NASSA users over the 8 years (sRR 1.32 [95% CI, 1.30–1.34], 1.89 [95% CI, 1.83–1.96], and 1.95 [95% CI, 1.89–2.00], respectively). While TCA users (sRR 1.02 [95% CI, 1.00–1.03]) and users of other antidepressants (e.g., trazodone, sRR 1.04 [95% CI, 0.99–1.10]) remained stable, MAOI use decreased (sRR 0.81 [95% CI, 0.64–1.02], although declining from a very low base prevalence) between 2012 and 2019. Distribution of antidepressant groups among all antidepressant users is provided in the Additional file 3: Table S4.

Table 2 shows that in both years, the proportions of women prescribed at least 1 antidepressant were much higher than for men, but rose for both sexes between 2012 and 2019, especially in men (sRR 1.34 [95% CI, 1.32–1.36] for men vs sRR 1.24 [95% CI, 1.23–1.26] for women). In both years, the prevalence of antidepressant users was higher among people aged 40 years and older (highest prevalence among people aged 40 to 64 in 2019 and highest among people aged 80 or older in 2012) than in the younger age group, but the highest increase in antidepressant use was seen for people aged 18 to 39 years (from 7.4% in 2012 to 11.2% in 2019; sRR of 1.49 [95% CI, 1.46–1.52]). In both years, the vast majority of antidepressant users were dispensed a single agent but the prevalence of people who were dispensed two or more antidepressants in a quarter increased markedly between 2012 and 2019 (from 1.0 to 1.6%; sRR 1.67 [95% CI, 1.62–1.72]).

**Table 2:** Antidepressants dispensed to residents aged ≥ 18 years in 2012 and 2019

	Second quarter 2012	Second quarter 2019	**Relative risk 2019 vs 2012 **(95% CI)	
	No. of patients (crude %)	No. of patients (crude %, **age-sex standardised**^a^** %)**		
			Crude	Age-sex stand
*Use of any antidepressant in population*				
Total	73,600/614,421 (12.0)	92,601/607,215 (15.3;** 15.2)**	1.27 (1.26–1.28)	1.27 (1.26–1.28)
Single AD	67,688/614,421 (11.0)	82,900/607,215 (13.7; **13.6**)	1.24 (1.23–1.25)	1.24 (1.23–1.25)
≥ 2 ADs	5912/614,421 (1.0)	9701/607,215 (1.6; **1.6**)	1.66 (1.61–1.71)	1.67 (1.62–1.72)
Sex				
Women	51,083/315,046 (16.2)	62,556/310,363 (20.2; **20.1**)	1.24 (1.23–1.26)	1.24 (1.23–1.26)
Men	22,517/299,375 (7.5)	30,045/296,852 (10.1; **10.1**)	1.35 (1.32–1.37)	1.34 (1.32–1.36)
Age groups (years)				
18–39	14,179/191,155 (7.4)	20,831/186,719 (11.2; **11.1**)	1.50 (1.47–1.53)	1.49 (1.46–1.52)
40–64	36,965/269,912 (13.7)	44,493/252,871 (17.6; **17.5**)	1.28 (1.27–1.30)	1.28 (1.27–1.30)
65–79	15,710/111,195 (14.1)	19,310/121,229 (16.0; **16.0**)	1.13 (1.11–1.15)	1.13 (1.11–1.16)
≥ 80	6746/42,159 (16.0)	7967/46,396 (17.2; **17.4**)	1.07 (1.04–1.11)	1.09 (1.05–1.12)
Type of antidepressant drug class				
SSRI	39,791/614,421 (6.5)	51,244/607,215 (8.4; **8.5**)	1.30 (1.29–1.32)	1.32 (1.30–1.34)
TCA	25,198/614,421 (4.1)	25,833/607,215 (4.3; **4.2**)	1.04 (1.02–1.06)	1.02 (1.00–1.03)
SNRI	5092/614,421 (0.8)	9470/607,215 (1.6; **1.6**)	1.88 (1.82–1.95)	1.89 (1.83–1.96)
NASSA	6865/614,421 (1.1)	13,279/607,215 (2.2; **2.2**)	1.96 (1.90–2.01)	1.95 (1.89–2.00)
MAOI	160/614,421 (0.0)	128/607,215 (0.0; **0.0**)	0.81 (0.64–1.02)	0.81 (0.64–1.02)
Others	2586/614,421 (0.4)	2700/607,215 (0.4; **0.4**)	1.06 (1.00–1.11)	1.04 (0.99–1.10)
Deprivation quintile^b^				
1 (most deprived)	15,599/95,057 (16.4)	20,109/95,467 (21.1; **21.0**)	1.28 (1.26–1.31)	1.28 (1.25–1.30)
2	15,120/106,030 (14.3)	19,099/104,903 (18.2; **18.2**)	1.28 (1.25–1.30)	1.27 (1.25–1.30)
3	13,594/114,450 (11.9)	16,890/112,059 (15.1; **15.1**)	1.27 (1.24–1.30)	1.27 (1.24–1.30)
4	16,065/154,800 (10.4)	19,081/150,633 (12.7; **12.7**)	1.22 (1.20–1.24)	1.22 (1.20–1.25)
5 (least deprived)	9856/110,532 (8.9)	12,122/107,279 (11.3; **11.2**)	1.27 (1.24–1.30)	1.26 (1.22–1.29)
Residence^b,c^				
Large urban area	16,470/118,366 (13.9)	20,278/115,689 (17.5; **17.5**)	1.26 (1.24–1.28)	1.26 (1.23–1.28)
Urban area	32,305/258,032 (12.5)	40,939/253,421 (16.2; **16.1**)	1.29 (1.27–1.31)	1.29 (1.27–1.31)
Accessible rural area	18,953/179,545 (10.6)	23,187/177,669 (13.1; **13.1**)	1.24 (1.21–1.26)	1.24 (1.22–1.27)
Remote rural area	2506/24,926 (10.1)	2897/23,562 (12.3; **12.4**)	1.22 (1.16–1.29)	1.24 (1.18–1.30)

^a^Direct age-sex standardization to the 2012 population

^b^Deprivation and residence missing for 33,552 (5.5%) people registered in the index quarter of 2012 and 36,874 (6.1%) in 2019

^c^Scottish Executive Urban–Rural Classification

Consistent with the overall trend, antidepressant use increased between 2012 and 2019 in all 5 deprivation groups and all groups of urban vs rural residence. However, in both years, the prevalence of antidepressant use was markedly higher among those living in the most versus least socio-economically deprived areas (16.4% vs 8.9% in 2012 and 21.1% vs 11.3% in 2019), and it was higher among residents of urban vs rural areas (13.9% vs 10.1% in 2012 and 17.5% vs 12.3% in 2019).

### Changes in long-term use of antidepressants between 2012 and 2019

Table 3 shows that among antidepressant users, the crude proportion of long-term users increased from 54.3 to 61.9% between 2012 and 2019 (sRR of 1.16 [95% CI, 1.15–1.17]). Twice as many antidepressant users were dispensed two or more antidepressants long term in 2019 compared to 2012 (2.0% in 2012 vs. 4.0% in 2019). The proportions of women prescribed antidepressants long term were higher than for men in both years, but rose for both sexes between 2012 and 2019 (sRR 1.17 [95% CI, 1.16–1.18] for women vs sRR 1.15 [95% CI, 1.13–1.17] for men).

**Table 3:** Long-term (> 2 years) use among antidepressant users in 2012 and 2019

	Second quarter 2012	Second quarter 2019	**Relative risk 2019 vs 2012 **(95% CI)	
	No. of patients (crude %)	No. of patients (crude %, **age-sex standardised**^a^** %)**		
			Crude	Age-sex stand
≥ 1 AD	39,984/73,600 (54.3)	57,361/92,601 (61.9; **63.1**)	1.14 (1.13–1.15)	1.16 (1.15–1.17)
≥ 2 AD	1480/73,600 (2.0)	3632/92,601 (4.0; **4.0**)	1.95 (1.84–2.07)	2.00 (1.88–2.13)
Sex				
Women	28,450/51,083 (55.7)	40,161/62,556 (64.2; **65.0)**	1.15 (1.14–1.16)	1.17 (1.16–1.18)
Men	11,534/22,517 (51.2)	17,200/30,045 (57.3, **58.7)**	1.12 (1.10–1.14)	1.15 (1.13–1.17)
Age groups (years)				
18–39	4632/14,179 (32.7)	8061/20,831 (38.7, **40.5)**	1.18 (1.15–1.22)	1.24 (1.20–1.28)
40–64	21,143/36,965 (57.2)	29,928/44,493 (67.3, **66.9)**	1.18 (1.16–1.19)	1.17 (1.16–1.18)
65–79	10,186/15,710 (64.8)	14,063/19,310 (72.8, **72.9)**	1.12 (1.11–1.14)	1.13 (1.11–1.14)
≥ 80	4023/6746 (59.6)	5309/7967 (66.6, **66.9)**	1.12 (1.09–1.15)	1.12 (1.09–1.15)
Long–term use among each antidepressant drug class				
SSRI	17,428/39,791 (43.8)	28,231/51,244 (55.1, **56.5)**	1.26 (1.24–1.28)	1.29 (1.27–1.31)
TCA	13,764/25,198 (54.6)	14,906/25,833 (57.7, **57.9)**	1.06 (1.04–1.07)	1.06 (1.04–1.08)
SNRI	2744/5092 (53.9)	5482/9470 (57.9, **58.0)**	1.07 (1.04–1.11)	1.08 (1.04–1.11)
NASSA	2560/6865 (37.3)	6082/13,279 (45.8, **45.5)**	1.23 (1.19–1.27)	1.22 (1.17–1.27)
MAOI	111/160 (69.4)	95/128 (74.2, **75.6)**	1.07 (0.93–1.24)	1.09 (0.95–1.25)
Others	1161/2586 (44.9)	1550/2700 (57.4, **57.3)**	1.28 (1.21–1.35)	1.28 (1.21–1.35)
Deprivation quintile^b^				
1 (most deprived)	8775/15,599 (56.3)	12,659/20,109 (63.0; **64.2**)	1.12 (1.10–1.14)	1.14 (1.12–1.16)
2	8478/15,120 (56.1)	11,970/19,099 (62.7; **63.9**)	1.12 (1.10–1.14)	1.14 (1.12–1.16)
3	7403/13,594 (54.5)	10,502/16,890 (62.2; **63.3**)	1.14 (1.12–1.16)	1.16 (1.14–1.19)
4	8599/16,065 (53.5)	11,864/19,081 (62.2; **63.3**)	1.16 (1.14–1.18)	1.18 (1.16–1.20)
5 (least deprived)	5133/9856 (52.1)	7375/12,122 (60.8; **61.8**)	1.17 (1.14–1.20)	1.19 (1.16–1.22)
Residence^b,c^				
Large urban area	9450/16,470 (57.4)	13,109/20,278 (64.6; **65.7**)	1.13 (1.11–1.15)	1.14 (1.13–1.16)
Urban area	17,533/32,305 (54.3)	25,170/40,939 (61.5; **62.9**)	1.13 (1.12–1.15)	1.16 (1.14–1.17)
Accessible rural area	10,158/18,953 (53.6)	14,279/23,187 (61.6; **62.5**)	1.15 (1.13–1.17)	1.17 (1.15–1.19)
Remote rural area	1247/2506 (49.8)	1812/2897 (62.5; **64.0**)	1.26 (1.20–1.32)	1.29 (1.22–1.35)

^a^Direct age-sex standardization to the 2012 population

^b^Deprivation and residence missing for 3366 (4.6%) antidepressant users in 2012 and 5300 (5.7%) in 2019

^c^Scottish Executive Urban–Rural Classification

In both years, long-term use was common among users of all antidepressant classes (ranging from 37.3 to 69.4% in 2012 and from 45.8 to 74.2% in 2019) and increased in all classes, most markedly among users of SSRIs (sRR 1.29 [95% CI, 1.27–1.31]), NASSAs (sRR 1.22 [95% CI, 1.17–1.27]), and other antidepressants (sRR 1.28 [95% CI, 1.21–1.35]). As for antidepressant use overall, the prevalence of long-term antidepressant use was higher among antidepressant users aged 40 years or older than among younger people, but it increased more among younger people (sRR 1.24 [95% CI, 1.20–1.28]) than for people aged 40 years or older (sRRs ranging from 1.12 [95% CI, 1.09–1.15] for people aged 80 or older to 1.17 [95% CI, 1.16–1.18] for people aged between 40 and 64).

The trend of long-term antidepressant use was generally similar across socioeconomic deprivation quintiles and across urban vs rural residence quartiles with the exception of a larger increase in long-term antidepressant users among residents in remote rural vs more accessible rural and urban areas (sRR 1.29 vs 1.17 to 1.14).

### Changes in potential high-risk use of antidepressants between 2012 and 2019

Table 4 shows that between 2012 and 2019, the prevalence of any high-risk use among antidepressant users decreased slightly (from a crude rate of 37.9 to 34.7%; sRR 0.93 [95% CI, 0.92–0.95]). Nevertheless, the total number of patients with any high-risk use of antidepressants increased between 2012 and 2019 from 27,861 to 32,131. In both years, approximately half of patients with any high-risk use triggered only one indicator (50.1% in 2012 and 54.2% in 2019), while the remainder triggered two or more.

**Table 4:** Potential high-risk use among antidepressant users in 2012 and 2019

	Second quarter 2012	Second quarter 2019	**Relative risk 2019 vs 2012** (95% CI)	
	No. of patients (crude %)	No. of patients (crude %, **age-sex standardised**^a^** %)**		
			Crude	Age-sex stand
*No. of potential high-risk use indicators triggered*				
Any	27,861/73,600 (37.9)	32,131/92,601 (34.7, **35.3**)	0.92 (0.90–0.93)	0.93 (0.92–0.95)
1	13,957/73,600 (19.0)	17,395/92,601 (18.8; **18.9**)	0.99 (0.97–1.01)	1.00 (0.98–1.02)
2	6300/73,600 (8.6)	7617/92,601 (8.2; **8.4**)	0.96 (0.93–0.99)	0.99 (0.95–1.02)
3	3343/73,600 (4.5)	3664/92,601 (4.0; **4.1**)	0.87 (0.83–0.91)	0.90 (0.86–0.94)
≥ 4	4261/73,600 (5.8)	3455/92,601 (3.7; **3.9**)	0.64 (0.62–0.67)	0.67 (0.64–0.70)
Sex				
Women	19,327/51,083 (37.8)	21,911/62,556 (35.0; **35.5)**	0.93 (0.91–0.94)	0.94 (0.92–0.95)
Men	8534/22,517 (37.9)	10,220/30,045 (34.0; **34.8)**	0.90 (0.88–0.92)	0.92 (0.90–0.94)
Age groups (years)				
18–39	3165/14,179 (22.3)	3653/20,831 (17.5; **17.9)**	0.79 (0.75–0.82)	0.80 (0.76–0.84)
40–64	11,193/36,965 (30.3)	11,714/44,493 (26.3; **26.1)**	0.87 (0.85–0.89)	0.86 (0.84–0.88)
65–79	9180/15,710 (58.4)	11,578/19,310 (60.0; **59.9)**	1.03 (1.01–1.04)	1.03 (1.01–1.04)
≥ 80	4323/6746 (64.1)	5186/7967 (65.1; **65.0)**	1.02 (0.99–1.04)	1.02 (0.99–1.04)
High-risk use among each antidepressant drug class				
SSRI	19,376/39,791 (48.7)	21,013/51,244 (41.0; **42.2)**	0.84 (0.83–0.85)	0.87 (0.85–0.88)
TCA	7375/25,198 (29.3)	8197/25,833 (31.7; **31.8)**	1.08 (1.06–1.11)	1.09 (1.06–1.11)
SNRI	2514/5092 (49.4)	4532/9470 (47.9; **47.2)**	0.97 (0.94–1.00)	0.96 (0.92–1.00)
NASSA	2846/6865 (41.5)	5616/13,279 (42.3; **41.3)**	1.02 (0.99–1.06)	1.00 (0.96–1.04)
MAOI	37/160 (23.1)	39/128 (30.5; **30.6)**	1.32 (0.90–1.94)	1.32 (0.92–1.91)
Others	1014/2586 (39.2)	1146/2700 (42.4; **38.9)**	1.08 (1.01–1.16)	0.99 (0.93–1.06)
Deprivation quintile^b^				
1 (most deprived)	5952/15,599 (38.2)	7027/20,109 (34.9; **35.8**)	0.92 (0.89–0.94)	0.94 (0.91–0.97)
2	5755/15,120 (38.1)	6545/19,099 (34.3; **35.0**)	0.90 (0.88–0.93)	0.92 (0.89–0.95)
3	5167/13,594 (38.0)	5855/16,890 (34.7; **35.0**)	0.91 (0.89–0.94)	0.92 (0.89–0.95)
4	6247/16,065 (38.9)	6914/19,081 (36.2; **36.8**)	0.93 (0.91–0.96)	0.95 (0.92–0.97)
5 (least deprived)	3705/9856 (37.6)	4235/12,122 (34.9; **34.8**)	0.93 (0.90–0.96)	0.92 (0.89–0.96)
Residence^b,c^				
Large urban area	6445/16,470 (39.1)	7102/20,278 (35.0; **35.7**)	0.90 (0.87–0.92)	0.91 (0.89–0.94)
Urban area	12,170/32,305 (37.7)	14,251/40,939 (34.8; **35.6**)	0.92 (0.91–0.94)	0.95 (0.93–0.96)
Accessible rural area	7163/18,953 (37.8)	8125/23,187 (35.0; **35.2**)	0.93 (0.90–0.95)	0.93 (0.91–0.96)
Remote rural area	1048/2506 (41.8)	1098/2897 (37.9; **38.4**)	0.91 (0.85–0.97)	0.92 (0.86–0.98)

^a^Direct age-sex standardization to the 2012 population

^b^Deprivation and residence missing for 3366 (4.6%) antidepressant users in 2012 and 5300 (5.7%) in 2019

^c^Scottish Executive Urban–Rural Classification

Stratification by age showed that high risk use decreased mainly among people aged < 65 years (sRR 0.80 for people aged 18 to 39 years [95% CI, 0.76–0.84] and sRR 0.86 [95% CI, 0.84–0.88] for people aged 40 to 64 years), while it remained stable among people aged 65 years or older (sRR 1.03 [95% CI, 1.01–1.04] for people aged 65 to 79 years and sRR 1.02 [95% CI, 0.99–1.04] for people aged 80 years or older). Among users of each antidepressant class, the proportion of people triggering any high-risk indicator increased markedly for MAOI users (sRR 1.32 [95% CI, 0.92–1.91]) during the observed period, decreased for SSRI users (sRR 0.87 [95% CI, 0.85–0.88]), and remained stable for TCAs, SNRIs, NASSAs, and other antidepressants (sRR 1.09 [95% CI, 1.06–1.11], sRR 0.96 [95% CI, 0.92–1.00], sRR 1.00 [95% CI, 0.96–1.04], and sRR 0.99 [95% CI, 0.93–1.06], respectively). However, the total number of patients triggering any indicator of potential high-risk use of antidepressant increased among all antidepressant user groups.

**Fig. 2:**
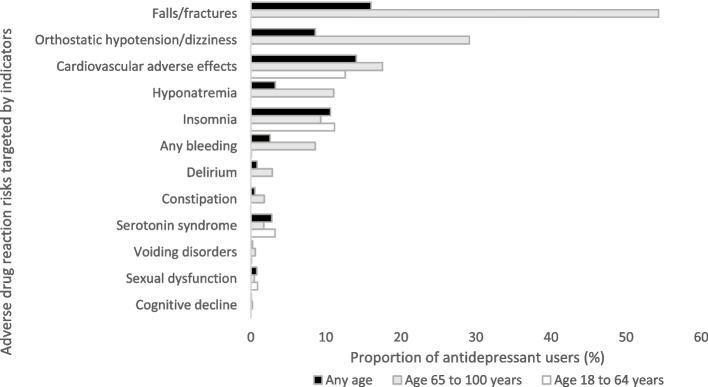
Proportion of antidepressant users triggering indicators targeting specific adverse drug reaction risks

High-risk use most commonly related to indicators targeting fall risk (16.0% of all antidepressant users), cardiovascular risks (14.1%), insomnia (10.6%), and risk of orthostatic hypotension (8.6%). Figure 2 shows that older and younger people differed substantially in terms of types of indicators triggered. For example, indicators targeting risk of fractures, orthostatic hypotension, hyponatremia, bleeding, and delirium were mostly relevant to people aged 65 years or older, whereas indicators targeting risk of cardiovascular events, insomnia, and serotonin syndrome were also relevant to younger people. Details on the prevalence of each individual potential high-risk use indicator in 2012 and 2019 are provided in the Additional file 3: Table S5, S6, and S7.

### Changes in the prevalence of potential deprescribing indications (PDIs) between 2012 and 2019

**Fig. 3:**
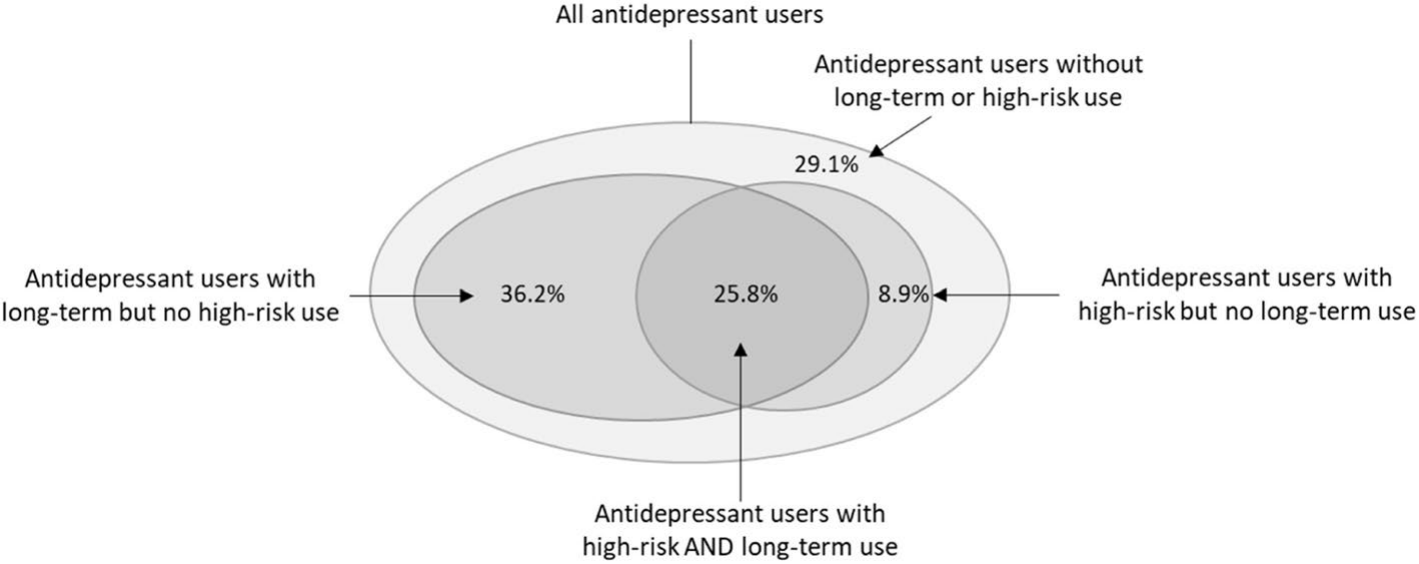
Venn diagram showing overlaps between long-term and potential high-risk use for primary analysis in 2019

Among all 92,601 antidepressant users in 2019, only 29.1% had no long-term or potential high-risk prescription, 36.2% had long-term but no potential high-risk prescription, 8.9% had potential high-risk prescription but no antidepressant long-term use, and 25.8% had both long-term and potential high-risk prescription (defined in this study as PDI) (Fig. 3). Between 2012 and 2019, the total number of patients with PDIs increased from 17,465 (23.7%) to 23,885 (25.8%), with sRR of 1.11 [95% CI, 1.10–1.13]. Details on the prevalence of PDIs stratified by patient variables are provided in the Additional file 3: Table S8.

### Patient characteristics associated with potential deprescribing indications in 2019

In the multivariate logistic regression analysis (Table 5), having potential deprescribing indications (PDIs) for antidepressants was most strongly associated with older age (65–79 versus 18–39; adjusted OR 14.12; 95% CI, 13.15–15.17) and the number of drugs dispensed (≥ 15 drugs versus 1–4 drugs; adjusted OR 7.37; 95% CI, 6.71–8.10). Women were slightly more likely to have PDIs than men (adjusted OR 1.07; 95% CI, 1.02–1.11). Compared to SSRIs, TCA use (higher dose) (adjusted OR 5.49; 95% CI, 5.02 to 6.01) and taking two or more antidepressants (adjusted OR 5.52; 95% CI, 5.20 to 5.85) were more likely to trigger PDIs, while other antidepressant use (in monotherapy) (SNRI, mirtazapine, other antidepressants e.g., trazodone) were less likely to trigger deprescribing indications compared to SSRI users. People living in more remote rural areas were less likely to have PDIs (adjusted OR 0.85; 95% CI, 0.76–0.95). After adjustment, socioeconomic status was not significantly associated with PDIs.

**Table 5:** Patient characteristics associated with PDIs (simultaneous long-term and potential high-risk use) in Q2 2019

**Variable (no. of patients)**^d^	**Odds ratio (95% CI) **crude	**Odds ratio (95% CI) **adjusted^a^
**Sex**		
Men (*n* = 23,633)	Reference	Reference
Women (*n* = 48,428)	1.17 (1.14–1.21)	1.07 (1.02–1.11)
**Age groups**		
18–39 (*n* = 17,390)	Reference	Reference
40–64 (*n* = 35,587)	2.82 (2.67–2.97)	2.17 (2.03–2.31)
65–79 (*n* = 13,453)	9.51 (8.99–10.05)	14.12 (13.15–15.17)
80–100 (*n* = 5631)	8.42 (7.88–8.98)	12.26 (11.23–13.37)
**Total no. of drugs dispensed Q2 2019**		
1 to 4 drugs (*n* = 34,165)	Reference	Reference
5 to 9 drugs (*n* = 24,322)	4.53 (4.35–4.71)	3.43 (3.27–3.59)
10 to 14 drugs (*n* = 10,251)	8.17 (7.80–8.56)	5.24 (4.94–5.57)
≥ 15 drugs (*n* = 3323)	12.72 (11.87–13.63)	7.37 (6.71–8.10)
**Antidepressant agent**^b^		
SSRI (*n* = 42,057) only	Reference	Reference
SNRI (*n* = 6518) only	1.25 (1.18–1.32)	0.95 (0.89–1.02)
TCA (≥ 50 mg) (*n* = 3388) only	7.04 (6.53–7.58)	5.49 (5.02–6.01)
Mirtazapine (low dose) (*n* = 3289) only	0.66 (0.60–0.72)	0.23 (0.21–0.26)
NASSA (high dose) (*n* = 5849) only	0.78 (0.73–0.84)	0.40 (0.37–0.43)
Other AD (*n* = 1824) only	0.90 (0.81–1.01)	0.39 (0.34–0.44)
Combined use of ≥ 2 ADs (*n* = 9136)	6.29 (6.00–6.59)	5.52 (5.20–5.85)
**Residence**^c^		
Large urban area (*n* = 17,336)	Reference	Reference
Urban area (*n* = 33,674)	0.95 (0.91–0.98)	0.90 (0.86–0.95)
Accessible rural area (*n* = 18,718)	0.93 (0.89–0.97)	0.86 (0.81–0.90)
Remote rural area (*n* = 2333)	1.01 (0.93–1.10)	0.85 (0.76–0.95)

^a^Adjusted for all variables shown in the table

^b^The list includes all antidepressants with potential deprescribing indications identified by the indicators (TCA < 50 mg only does not trigger any indicator)

^c^Scottish Executive Urban–Rural Classification

^d^Number of patients included in the multivariate analysis

### Sensitivity analyses

Restricting the definition of long-term use to continuous use without grace periods in SA1, the proportion of long-term use in 2019 decreased from 61.9 to 48.8% but the proportionate increase in long-term use between 2012 and 2019 was more pronounced (sRR 1.30 [95% CI, 1.29–1.32]).

When we restricted high-risk use to instances where antidepressants were co-prescribed with ≥ 2 fall-risk increasing drugs (FRIDs) in SA 2 (as opposed to ≥ 1 FRID in primary analysis), the prevalence of patients at risk from this specific indicator in 2019 reduced from 51.4 to 28.2%, although there was only minimal reduction in the proportion of people triggering ≥ 1 potential high-risk indicator in 2019 (from 34.7 to 32.6%). When we restricted high-risk use to instances where patients were co-prescribed ≥ 2 drugs increasing the risk of Torsades de Point in SA3 (as opposed to ≥ 1 drug in primary analysis), the prevalence of patients at risk from this specific indicator in 2019 reduced from 12.2 to 4.9%, but again with minimal reduction in the proportion of people triggering ≥ 1 potential high-risk indicator in 2019 (from 34.7 to 30.3%). When we restricted high risk use to indicators with median 8 and 9 in SA4 (which also excluded the falls risk and Torsade de Point indicators in SA2 and SA3), the prevalence of patients with antidepressant potential high-risk prescribing in 2019 decreased from 34.7 to 9.4%. Among all antidepressant users in 2019, 6.5% had both long-term use (without grace periods (SA1)) and potential high-risk prescription (taking in account only indicators with the highest ratings (8 and 9) (SA4)). The results of the sensitivity analysis for 2012 are provided in Additional file 3: Table S9.

## Discussion

### Summary of findings

Between 2012 and 2019, antidepressant use in adult residents of two Scottish regions increased by more than a quarter (sRR 1.27) from 12.0 to 15.3%. While antidepressant users were mostly older (77.5% were ≥ 40 years in 2019), the largest relative increase (sRR of 1.49) was seen in younger adults aged 18–39 years. Antidepressant use was nearly twice as high among residents in the most socially deprived (21.0%) versus least deprived (11.3%) areas in 2019. Among antidepressant users, long-term use (> 2 years) increased from 54.3% in 2012 to 61.9% in 2019 (sRR 1.16), while potential high-risk use decreased from 37.9% in 2012 to 34.7% in 2019 (sRR 0.93). Nevertheless, the absolute number of people with potential high-risk use of antidepressants was higher in 2019 vs 2012 (32,131 vs 27,861). Potential deprescribing indications (PDIs) (defined in this study as simultaneous long-term and potential high-risk use) increased from 23.7 to 25.8% (sRR 1.11). When we applied stricter definitions of long-term and potential high-risk use in sensitivity analyses vs primary analyses, the prevalence of long-term antidepressant use in 2019 was somewhat lower (48.8% vs 61.9%), whereas the prevalence of PDIs (6.5% vs 25.8%) was substantially lower. The presence of PDI was most strongly associated with increasing age and with more drugs taken concomitantly, but also with the use of TCAs (at doses ≥ 50 mg) and concomitant use of 2 or more antidepressants compared to the use of SSRIs only.

### Comparison to literature

To the best of our knowledge, there are no directly comparable investigations of potential high-risk use of antidepressants. However, our findings are consistent with previous studies demonstrating increased use of antidepressants in general and of increasing long-term use in particular [5–7, 9, 12, 34, 35]. For example, in the Swiss population in 2019, 57.4% of antidepressant users were long-term users [6]. Similarly, in a prospective cohort study in UK general practice in 2012, the prevalence of long-term antidepressant use was 47.1% [9], while our findings show slightly higher prevalences of long-term use in both years (54.3% in 2012 and 61.9% in 2019). Twice as many women were prescribed at least one antidepressant in 2019, a pattern that has repeatedly been reported in other studies [5, 6, 36]. Socio-economic deprivation is a known risk factor for depression [37], which is consistent with our finding of a higher prevalence of antidepressant use in the socio-economically deprived population.

Current clinical guideline recommendations and general consensus is that SSRIs, SNRIs, and mirtazapine are first-line or preferred antidepressants, mainly due to their favorable safety profile in comparison to other antidepressants [29, 38, 39]. This may at least partially explain why the use of these antidepressants has particularly increased between 2012 and 2019. Increased use of mirtazapine has also been observed in a study conducted in Spain, Germany, Denmark, and Sweden [40] and may also be attributed to clinical guideline recommendations advocating combination therapy with mirtazapine for patients who do not respond to initial antidepressant treatments with SSRIs and SNRIs [1, 3]. In addition, an increasing use of SNRIs and mirtazapine for indications other than depression, such as chronic pain and insomnia, may also be a contributing factor [41–43]. For example, some resources consider off-label use of mirtazapine as a safer alternative to benzodiazepines in the treatment of insomnia [44, 45].

Our results show a high proportion of antidepressant and long-term use among older adults, similar to studies from other countries [6, 46, 47]. For example, in the Swiss population in 2019, 56.1% of long-term antidepressant users were older than 60 years compared to 33.8% of long-term users being 65 years or older in this study.

While there are no directly comparable investigations of potential deprescribing indications of antidepressants, our findings in this population-based database study are consistent with a prospective cohort study in UK general practice, where GP review of antidepressant use revealed that antidepressants could be stopped, reduced, or switched (deprescribed) in almost one-quarter (23.2%) of antidepressant users [9].

### Strengths and limitations

To our knowledge, this is the first study to investigate potential deprescribing indications for antidepressants using validated explicit criteria [24]. Key methodological strengths include the large population-based sample, the measurement of antidepressant use based on pharmacy-dispensed prescriptions, enabling reliable comparisons over time across a number of measures, as well as stratified analysis by gender, age, socioeconomic deprivation, and residency in rural vs urban areas.

Our study has a few limitations, which may affect the levels of long-term and potential high-risk use measured. Unavailability of over the counter dispensed drugs that may interact with antidepressants (e.g., non-steroidal anti-inflammatory drugs and antihistamines), unavailability of ambulatory care diagnoses (and use of dispensed drugs or hospital diagnoses as proxies), and unavailability of dosing instructions (and use of drug strength as a proxy for daily dosing of TCAs) decrease the observed levels of potential high-risk use (as defined by this validated indicator set). In contrast, our definition of combined use of antidepressants with interacting drugs (dispensation in the same 3-month period) may overestimate the prevalence of potential high-risk drug-drug interactions. Although these factors may influence the precision of period-prevalence estimates, comparisons between the years 2012 and 2019 remain valid since any measurement errors affected both years equally.

While our analysis was based on data from two Scottish health boards, we cannot exclude that the prevalence in other regions may differ. Nonetheless, Tayside and Fife are representative of Scotland in terms of age and socioeconomic deprivation [48].

Although we assessed prevalences of antidepressant use and their long-term and/or high-risk use at a single point in time in 2012 and 2019, there is minimal seasonal variation in antidepressant dispensing [30], and for all comparisons between years, we used the same time points.

### Implications for clinical practice and research

Our results confirm the global trend of increasing antidepressant use and their prevalent long-term prescriptions. Long-term use may be a consequence of few discontinuations attempts in primary care, which may be due to fear of relapse and withdrawal effects [22, 49]. Given that longer duration of use may be associated with increased severity and duration of withdrawal symptoms [21], timely identification of PDIs is clearly important. Lack of awareness of the potential risks associated with long-term antidepressant use could also be one of the reasons for few discontinuation attempts [23]. Our findings that potential high-risk use most commonly relates to increased risk of falls/fractures, orthostatic hypotension, cardiovascular adverse effects, insomnia, and bleeding emphasizes that increased risk awareness is particularly relevant in frail, older people. Although the prevalence of any high-risk use among antidepressant users seemed to decrease, the higher absolute number of patients indicates a greater absolute burden on the healthcare system due to increased risks of adverse drug events.

The indicator set applied here has been developed to enable continuous monitoring of potentially inappropriate use of antidepressants at population level (e.g., for clinical surveillance or research purposes), as a basis for (computerized) decision support and for case finding (e.g., to identify patients in need of a medication review) [50, 51]. While most randomized trials on deprescribing antidepressants target patients with long-term use [52], our analysis highlights the potential importance of also considering high-risk use of antidepressants as a reason to critically review their continued use. This study has demonstrated that most (but not all) indicators in the set can be operationalized in administrative data sources and that implemented indicators can detect changes in long-term and potential high-risk antidepressant use and highlight priorities for improvement. Higher precision in the measurement of period prevalence of potential high-risk use will be achievable in data sources that additionally include ambulatory care diagnoses and dosing instructions.

When all indicators were implemented, we found that 1 in 4 antidepressant users have potential deprescribing indications and may require review, while restriction to indicators with the highest ratings in the preceding expert consensus study yielded substantially fewer antidepressant users with potential deprescribing indications (1 in 15). Although all indicators were validated as scenarios in which a review of antidepressant use was deemed “necessary” (see definition here [24]), focusing on indicators of particular importance may be a pragmatic implementation strategy in resource restricted settings.

Although all criteria used in this study were systematically developed using evidences synthesis and expert consensus [24], it is important to note that explicit criteria applied to routine data sources, as this study has done, can only highlight *potential* deprescribing indications. Decisions to stop or alter treatment in individual patients requires careful consideration of the benefits and risks of continuing vs altering antidepressant treatment (and/or co-medication increasing risk of adverse antidepressant effects) by clinicians and their patients. Empirical validation studies are required in order to examine the performance of the indicator set (sensitivity and specificity) in identifying *actual* deprescribing opportunities and to guide any indicator adaptation and optimization.

## Conclusions

While antidepressants have an essential role in the treatment of severe forms of depression and anxiety, we found that long-term and potential high-risk use is widespread and potential deprescribing indications (PDIs) are increasing, suggesting a need for effective deprescribing interventions. This study demonstrates that the indicator set applied here may be used as an instrument to monitor potentially inappropriate use of antidepressants at population level and to identify patients with PDIs, who might benefit from a critical review of antidepressant continuation. As antidepressant use continues to increase internationally, these indicators may encourage comparative analyses of the prevalence of deprescribing indications in other settings.

## Supplementary Information

Additional file 1: Fig. S1. Operationalisation of long-term us.

Additional file 2: Tables S1-S3. Table S1. Original and operationalised indicators for high-risk prescribing. Table S2. Specification of exposure to comorbidity. Table S3. Specification of exposure to comedication.

Additional file 3: Tables S4-S9. Table S4. Distribution of antidepressant groups among all antidepressant users. Table S5. Prevalence of each potential high-risk use indicator in 2012 and 2019. Table S6. Proportion of antidepressant users triggering indicators targeting specific adverse drug reaction risks (2012). Table S7. Proportion of antidepressant users triggering indicators targeting specific adverse drug reaction risks (2019). Table S8. PDIs among antidepressant users in 2012 and 2019. Table S9. Sensitivity analysis.

## Data Availability

The data underlying this article are available in the article and in its online supplementary material. Further supporting materials are available upon request.

## Abbreviations

PDIs: Potential deprescribing indications
NHS: National Health Service
BNF: British National Formulary
HIC: Health Informatics Centre
TCA: Tricyclic antidepressants
SSRI: Selective serotonin-reuptake inhibitors
SNRI: Selective serotonin-norepinephrine reuptake inhibitors
NASSA: Noradrenergic and specific serotonergic antidepressants
MAOIs: Monoamine oxidase inhibitors
AD: Antidepressant
CI: Confidence interval
SA: Sensitivity analysis
SD: Standard deviation
sRR: Standardized relative risk
FRIDs: Fall-risk increasing drugs
TdP: Torsades de Point
