# Supplementary material for: Potential deprescribing indications for antidepressants between 2012 and 2019: repeated cross-sectional analysis in two Scottish health boards
Source: BMC Med. 2024 Sep 11;22:378. doi: 10.1186/s12916-024-03584-9 (PMC11389559; doi:10.1186/s12916-024-03584-9)

Additional file 1

To identify long-term use for individuals who were dispensed at least one antidepressant in each index quarter in 2012 and 2019, we considered the 8 preceding quarters and counted the number of quarters in which they had at least one antidepressant prescription. Allowing for a grace period of one quarter, there had to be at least one antidepressant prescription every second quarter dispensed to qualify as long-term use. In order to determine exposure status in Q2 2010 and Q2 2017, we also considered antidepressant use in the previous quarters.

Figure S1: Operationalisation of long-term use - Minimal requirements for dispensing of antidepressants to qualify as “long-term use”. In case there was no dispensing of antidepressants in Q2 of 2010 (highlighted by a red circle), we also considered the previous quarter (consistent with a grace period of one quarter). The same principal applied to the assessment of long-term antidepressant use in Q2 2019.

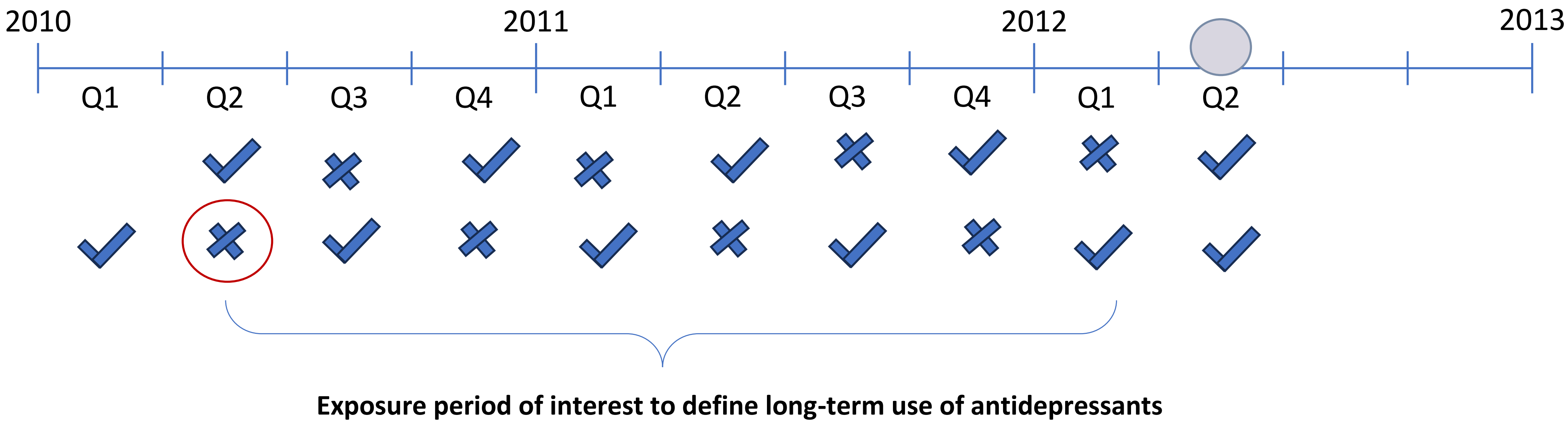

Supplement: Supplementary file 1 — Additional file 1: Fig. S1. Operationalisation of long-term us. [file 12916_2024_3584_MOESM1_ESM.pdf]
