# Supplementary material for: Potential deprescribing indications for antidepressants between 2012 and 2019: repeated cross-sectional analysis in two Scottish health boards
Source: BMC Med. 2024 Sep 11;22:378. doi: 10.1186/s12916-024-03584-9 (PMC11389559; doi:10.1186/s12916-024-03584-9)
Supplement: Supplementary file 2 — Additional file 2: Tables S1-S3. Table S1. Original and operationalised indicators for high-risk prescribing. Table S2. Specification of exposure to comorbidity. Table S3. Specification of exposure to comedication. [file 12916_2024_3584_MOESM2_ESM.pdf]

Additional file 2: Operationalisation of indicators of potential high-risk use

Table S1: Original and operationalised indicators for high-risk prescribing

Table S2: Specification of exposure to comorbidity

Table S3: Specification of exposure to comedication

Table S1: Original and operationalised indicators for high-risk prescribing

High-risk prescribing indicators (original wording)	Median	Operationalization in the data set
Cardiovascular adverse effects		
**C1.** Prescribed SNRI or TCA (in doses ≥50 mg/day)[A] or tranylcypromine[B] - and patient has a history of chronic heart failure.	8	**Yes** – Dispensed SNRI or TCA (dispensed strength ≥50 mg) or tranylcypromine and previous hospital admission for heart failure (in the 2 years prior to the index quarter)
**C2.** Prescribed TCA (in doses ≥50 mg/day) - and patient has a history of ischemic heart disease.	8	**Yes** – Dispensed TCA (dispensed strength ≥50 mg) and previous hospital admission for ischemic heart disease (in the 2 years prior to the index quarter)
**C3.** Prescribed >20mg citalopram or >10mg escitalopram daily - and patient is aged ≥65 years (risk of QTc prolongation).	7	**Yes** – Dispensed citalopram (dispensed strength >20mg) or escitalopram (dispensed strength >10mg) and aged ≥65 years
**C4.** Prescribed citalopram, escitalopram - and patient has long QT-Syndrome or is at risk of long QT-syndrome (e.g., (advanced) chronic heart failure, ischemic heart disease, myocardial hypertrophy, bradyarrhythmias or an ongoing risk of hypokalaemia).	9	**Yes** – Dispensed citalopram or escitalopram and previous hospital admission for heart failure, myocardial hypertrophy or bradycardia (atrioventricular block) (in the 2 years prior to the index quarter) or a recent hospital admission for myocardial infarction (in the year prior to the index quarter)
**C5.** Prescribed citalopram, escitalopram, TCA (in doses ≥50 mg/day), maprotiline, mianserin - and patient is co-prescribed ≥1 further drug with any risk of TdP.	7	**Yes** - Dispensed citalopram or escitalopram or TCA (dispensed strength ≥50 mg) or maprotiline or mianserin and also dispensed ≥1 drug on the credible meds list (Woosley RL et al. CredibleMeds; QTdrugs List) in the three months (from April 1st to June 30th) prior to the index date
Prescribed TCA (in doses ≥50 mg/day) or SNRI or bupropion or tranylcypromine[2] - and patient has developed tachycardia.	8	**No**
**C6.** Prescribed fluoxetine, paroxetine or bupropion - and patient is co-prescribed metoprolol or propranolol (risk of bradycardia).	7	**Yes** – Dispensed fluoxetine or paroxetine or buproprion and also dispensed metoprolol or propranolol in the three months prior to the index date
Prescribed SNRI or TCA (in doses ≥50 mg/day) or bupropion or tranylcypromine - and patient has uncontrolled hypertension.	8	**No**

**C7.** Prescribed SNRI or TCA (in doses ≥50 mg/day) or bupropion or tranylcypromine - and achieving hypertension control requires ≥3 antihypertensive drugs.	8	**Yes** – Dispensed SNRI or TCA (dispensed strength ≥50 mg) or tranylcypromine and also dispensed ≥3 antihypertensive drugs (ACE-Inhibitor/ Angiotensin Receptor Blocker/Aliskiren, Calcium Channel Blocker, Diuretics, Beta-adrenoceptor blocking drugs, Alpha blocker – antihypertensives, Centrally acting antihypertensives) in the three months prior to the index date
**Orthostatic hypotension (OH)/dizziness**		
Prescribed TCA (in doses ≥50 mg/day) or trazodone or tranylcypromine - and patient has developed persistent OH/dizziness under treatment.	8	**No**
Prescribed SSRI or SNRI or mirtazapine - and patient is aged ≥65 years and has developed persistent OH/dizziness under treatment.	8	**No**
**OH1.** Prescribed TCA (in doses ≥50 mg/day) or trazodone or tranylcypromine - and patient is aged ≥65 years and co-prescribed ≥1 further drug with known blood pressure lowering effect (e.g., α-blockers, β-blockers, nitrates, SGLT-inhibitors, levodopa, antipsychotics).	7	**Yes -** Dispensed TCA (dispensed strength ≥50 mg) or trazodone or tranylcypromine, aged ≥65 years and also dispensed ≥1 further drug with known blood pressure lowering effect (ACE-inhibitor/Angiotensin receptor blocker/Aliskiren, Calcium Channel Blocker, Diuretics, β-blockers, α-blockers, Nitrates, Centrally acting antihypertensives, Atypical antipsychotics) in the three months prior to the index date
**OH2.** Prescribed SSRI or SNRI or mirtazapine - and patient is aged ≥65 years and co-prescribed ≥2 further drugs with blood pressure lowering effect (e.g., α-blockers, β-blockers, nitrates, SGLT-inhibitors, levodopa, antipsychotics).	7	**Yes –** Dispensed SSRI or SNRI or mirtazapine, aged ≥65 years and also dispensed ≥2 further drugs with known blood pressure lowering effect in the three months prior to the index date
**Falls and fall-related injuries**		
F1. Prescribed any antidepressant - and patient is aged ≥65 years and co-prescribed ≥1 further fall-risk-increasing drug.	7	**Yes –** Dispensed SSRI or SNRI or TCA (dispensed strength ≥50 mg) or mirtazapine or trazodone, aged ≥65 years and also dispensed ≥1 fall-risk increasing drug (according to Seppala LJ et al. STOPPFall 2021) (Alpha blocker as antihypertensive and for prostate hyperplasia, Antipsychotics, Antihistamines, Benzodiazepines, Z-drugs, Overactive bladder and urge incontinence medications, Opioids, Antiepileptics, Centrally acting antihypertensives, Diuretics, Vasodilators in cardiac disease,

		Anticholinergic drugs with ACB score 2 or 3) in the three months prior to the index date
F2. Prescribed any antidepressant - and patient has a history of fall.	8	**Yes** – Dispensed SSRI or SNRI or TCA (dispensed strength ≥50 mg) or mirtazapine or trazodone, aged ≥65 years and previous hospital admission for falls (in the 2 years prior to the index quarter)
F3. Prescribed any antidepressant - and patient has cognitive impairment or dementia.	7	**Yes** – Dispensed SSRI or SNRI or TCA (dispensed strength ≥50 mg) or mirtazapine or trazodone, aged ≥65 years and also dispensed antidementia drugs (donepezil, galantamine, idebenone, memantine, rivastigmine – in the 2 years prior to the index quarter) or previous hospital admission for dementia (in the 2 years prior to the index quarter)
F4. Prescribed any antidepressant - and patient has a history of stroke and co-prescribed ≥1 further fall-risk-increasing drug.	8	**Yes** - Dispensed SSRI or SNRI or TCA (dispensed strength ≥50 mg) or mirtazapine or trazodone, aged ≥65 years and previous hospital admission for ischemic or hemorrhagic stroke (in the 2 years prior to the index quarter) and also dispensed ≥1 further fall-risk-increasing drug in the three months prior to the index date
**Cognitive decline & delirium**		
**CD1.** Prescribed anticholinergic antidepressant opipramol or other TCA (in doses ≥50 mg/day) or paroxetine - and patient has cognitive impairment or dementia.	8	**Yes** – Dispensed TCA (dispensed strength ≥50 mg) or maprotiline or paroxetine and also dispensed antidementia drugs (donepezil, galantamine, idebenone, memantine, rivastigmine – in the 2 years prior to the index quarter) or previous hospital admission for dementia (in the 2 years prior to the index quarter)
**D1.** Prescribed anticholinergic antidepressant opipramol or other TCA (in doses ≥50 mg/day) or paroxetine - and patient has a history of delirium and co-prescribed ≥1 further drug known to induce delirium (e.g., benzodiazepines, opioids, antihistamines, diuretics).	8	**Yes** – Dispensed TCA (dispensed strength ≥50 mg) or maprotiline or paroxetine and previous hospital admission for delirium (in the last 2 years prior to the index quarter) and also dispensed ≥1 further drug known to induce delirium (GABA-agonists, Opioids, Diuretics, Glucocorticoids or Anticholinergic drugs with ACB score 2 or 3) in the three months prior to the index date

**D2.** Prescribed anticholinergic antidepressant opipramol or other TCA (in doses ≥50 mg/day) or paroxetine - and patient is aged ≥65 years and co-prescribed ≥2 further drugs known to induce delirium (e.g., benzodiazepines, opioids, antihistamines, diuretics).	9	**Yes** - Dispensed TCA (dispensed strength ≥50 mg) or maprotiline or paroxetine, aged ≥65 years and also dispensed ≥2 further drugs known to induce delirium in the three months prior to the index date
Serotonin syndrome		
**S1.** Prescribed tranylcypromine - and patient is co-prescribed ≥1 further serotonergic drug (e.g., tramadol, fentanyl, triptans, metoclopramide, SSRI, SNRI, TCA).	7	**Yes** – Dispensed tranylcypromine and also dispensed ≥1 further serotonergic drug (see table S3) in the three months prior to the index date
**S2.** Prescribed SSRI or SNRI or TCA (in doses ≥50 mg/day) - and patient is co-prescribed ≥2 further serotonergic drugs other than tranylcypromine (e.g., tramadol, fentanyl, triptans, metoclopramide, another serotonergic antidepressant).	8	**Yes –** Dispensed SSRI or SNRI or TCA (dispensed strength ≥50 mg) and also dispensed ≥2 further serotonergic drugs (see **Table S3**) in the three months prior to the index date
Gastrointestinal bleeding		
**G1.** Prescribed SSRI or SNRI - and patient is aged ≥65 years and co-prescribed a single of the following without GI-protection: antiplatelet, anticoagulant, NSAID.	7	**Yes** – Dispensed SSRI or SNRI, aged ≥65 years and also dispensed ≥1 drug with a risk of gastrointestinal bleeding (antiplatelets, anticoagulant, NSAID) and no PPI in the three months prior to the index date
**G2.** Prescribed SSRI or SNRI - and patient is aged ≥65 years and co-prescribed ≥2 of the following: antiplatelet, anticoagulant, NSAID (regardless of GI-protection).	8	**Yes -** Dispensed SSRI or SNRI, aged ≥65 years and also dispensed ≥2 drugs with a risk of gastrointestinal bleeding (antiplatelets, anticoagulant, NSAID) in the three months prior to the index date
**G3.** Prescribed SSRI or SNRI - and patient has at least one risk factor for GI bleeding (history of peptic ulcer disease, GI-bleeding or haemophilia) and co-prescribed ≥1 of the following: antiplatelet, anticoagulant, NSAID (regardless of GI-protection).	9	**Yes** – Dispensed SSRI or SNRI and has a previous hospital admission for peptic ulcer disease or gastrointestinal bleeding (in the 2 years prior to the index quarter) and also dispensed ≥1 drug with a risk of gastrointestinal bleeding (antiplatelets, anticoagulant, NSAID) in the three months prior to the index date
Bleeding		
**B1.** Prescribed SSRI - and patient has a history of a bleeding event and co-prescribed ≥1 of the following: anticoagulant or antiplatelet.	8	**Yes** – Dispensed SSRI and has a previous hospital admission for a non-gastrointestinal bleeding (in the 2 years prior to the index quarter) and also dispensed ≥1 drug with a risk of bleeding (anticoagulant or antiplatelet) in the three months prior to the index date

**B2.** Prescribed SSRI - and patient has at least one risk factor for intracranial bleeding (aged ≥65 years, history of stroke, history of dementia) and co-prescribed ≥1 of the following: anticoagulant or antiplatelet.	7	**Yes** – Dispensed SSRI and has a previous hospital admission for hemorrhagic stroke or for dementia (in the last 2 years prior to the index quarter) and also dispensed ≥1 drug with a risk of bleeding (anticoagulant or antiplatelet)- in the three months prior to the index date
**Constipation**		
Prescribed anticholinergic antidepressant opipramol or other TCA (in doses ≥50 mg/day) or paroxetine - and patient has persistent constipation.	7	**No**
**C01.** Prescribed anticholinergic antidepressant opipramol or other TCA (in doses ≥50 mg/day) or paroxetine - and patient is aged ≥65 years and co-prescribed ≥2 further drugs known to have constipating effects (e.g., calcium antagonists, opioid, antihistamines, antipsychotics).	8	**Yes -** Dispensed TCA (dispensed strength ≥50 mg) or maprotiline or paroxetine, aged ≥65 years and also dispensed ≥2 further drugs known to have constipating effects (Opioids, Calcium Channel Blocker, Anticholinergic drugs with ACB score 2 or 3) in the three months prior to the index date
**Hyponatremia**		
Prescribed any antidepressant - and patient has developed hyponatremia (<130 mmol/l) under treatment without being treated with a diuretic.	7	**No**
**H1.** Prescribed SSRI or SNRI - and patient is aged ≥65 years and co-prescribed ≥2 further drugs known to cause hyponatremia (e.g., (thiazide) diuretics, antipsychotics, anticonvulsants, proton pump inhibitors).	8	**Yes** – Dispensed SSRI or SNRI, aged ≥65 years and also dispensed ≥2 further drugs known to cause hyponatremia (see **Table S3**) in the three months prior to the index date
**Hepatic injury**		
Prescribed agomelatine - and patient has developed elevated serum transaminase levels (>3 times the upper normal range) under treatment.	9	**No**
Prescribed agomelatine - and patient has hepatic impairment (i.e., cirrhosis or active liver disease).	8	**No**
**Voiding disorders**		
**VD1.** Prescribed anticholinergic antidepressant opipramol or other TCA (in doses ≥50 mg/day) or paroxetine - and patient has a history of voiding disorders (e.g., urinary retention or benign prostatic hyperplasia) or has developed urinary retention under treatment.	7	**Yes** – Dispensed TCA (dispensed strength ≥50 mg) or maprotiline or paroxetine and also dispensed ≥1 drug for benign prostatic hyperplasia or urinary retention (see **Table S3**) (in the six months prior to the index date)

**Glaucoma**		
Prescribed anticholinergic antidepressant opipramol or other TCA (in doses ≥50 mg/day) or paroxetine - and patient has a history of angle closure glaucoma or has developed angle closure glaucoma under treatment.	8	**No**
**Sleep disturbances/agitation**		
**I1.** Prescribed SSRI or SNRI or MAOI or bupropion - and patient has persistent sleeping disturbances (e.g., insomnia, restless leg syndrome) or is experiencing agitation.	7	**Yes** – Dispensed SSRI or SNRI or MAOI or bupropion and also dispensed ≥1 hypnotic (see **Table S3**) in the three months prior to the index date
**Sexual dysfunction**		
**SD1.** Prescribed SSRI or SNRI - and patient has developed sexual dysfunction.	8	**Yes** – Dispensed SSRI or SNRI and also dispensed ≥1 drug for erectile dysfunction (see **Table S3**) in the three months prior to the index date

A: It cannot be excluded that low-dose TCAs also have significant adverse effects, as evidence on the safety of low-dose TCAs is sparse; B: Especially when co-administered with tyramine-containing food. *Abbreviations:* SSRI = Selective serotonin-reuptake inhibitors; SNRI = Selective serotonin-norepinephrine reuptake inhibitors; TCA = Tricyclic antidepressant; TdP = Torsades de pointes; NSAID = Nonsteroidal anti-inflammatory drugs; GI = Gastrointestinal; MAOI = Monoamine oxidase inhibitors.

Table S2: Specification of exposure to comorbidity

Disease/Comorbidity	Definition (ICD-10 Codes)
Heart Failure	I110, I130, I132, I500, I501, I509
Ischemic Heart Disease	I20x, I21.x, I22.x, I23.x, I24.x, I25.x
Myocardial Infarction	I21, I22
Myocardial Hypertrophy	I42.1, I42.2
Bradyarrhythmias	I44.1, I44.2, I44.3, I45.8
History of Fall	Hospital admission was counted as fall-related, if:<br>- Main condition was one of the ICD-10-Codes of category 1 **OR**<br>- Main condition was one of the ICD-10-Codes of category 2 AND one of the ICD-10-Codes of category 4 was a secondary condition **OR**<br>- Main condition was one of the ICD-10-Codes of category 2 AND the patient was ≥ 65 years (category 5) **OR**<br>- Main condition was one of the ICD-10-Codes of category 3 AND one of the ICD-10-Codes of category 4 was a secondary condition AND the patient was ≥ 65 years (category 5)<br>If one of the following ICD-10-Codes V01-V99 (transportation accidents) has also been coded, the case was excluded.<br>**Categories:**<br>- <u>Category 1</u> (“strong” ICD-10-Codes: most important/frequent fractures: hip, femur, wrist, cranium): S00.-, S01.-, S05.-, T00.0, T01.0, S02.-, T02.0, S06.-, S03.4, S03.5, T03.0, S09.0, S04.-, S07.-, S03.0, S12.-, S22.-, S32.-, T08.0, T08.1, T02.1, T02.7, S42.-, S52.-, S62.-, T02.2, T02.4, T02.6, T10.0, T10.1, S40.7, S40.8, S40.9, S50.7, S50.8, S50.9, S60.7, S60.8, S60.9, T00.2, T11.0, S72.-, S82.-, S92.-, T02.3, T02.5, T12.0, T12.1, S70.0, S70.7, S70.8, S70.9, S80.7, S80.8, S80.9, S90.7, S90.8, S90.9, T00.3, T00.6, T13.0, S93.0, T02.8, T02.9, T14.2<br>- <u>Category 2</u> (“weaker” ICD-10-Codes: superficial injuries, open wounds, other fractures): S10.-, S11.-, S20.-, S21.-, S30.-, S31.-, T00.1, T01.1, T09.0, T09.1, S14.-, S15.-, S17.-, S24.-, S25.-, S28.0, S34.-, S35.-, S38.0, S38.1, T04.0, T04.1, T04.7, T06.0, T06.1, T09.3, T09.4, S41.-, S51.-, S61.-, T01.2, T01.6, T11.1, S40.0, S60.0, S60.1, S60.2, S50.0, S50.1, S43.0, S43.1, S43.2, S43.3, S53.0, S53.1, S63.0, S63.1, S63.2, T03.2, T03.4, S81.-, S91.0, S91.7, S90.0, S90.1, S90.2, S90.3, S70.1, S80.0, S80.1, S73.0, S70.0<br>- <u>Category 3</u> (unspecific ICD-10-Codes: other injuries, unspecified injury, other accident): S13.1, S13.2, S13.3, S23.1, S23.2, S33.1, S33.2, S33.3, T03.1, T09.2, S13.4, S13.5, S13.6, S16.-, S23.3, S23.4, S23.5, S33.5, S33.6, S33.7, S26.-, S27.-, S36.-, S37.-, T06.5, S43.4, S43.5, S43.6, S43.7, S46.-, S53.4, S56.-, S63.5, S63.6, S63.7, S66.-, T03.4, T11.2, S44.-, S45.-, S47, S54.-, S55,-, S57.-, S64.-, S65.-, S67.-, T04.2, T04.7, T04.4, T11.3, T11.4, S71.-, S91.3, S91.1, S91.2, T01.3, T13.1, S83.0, S83.1, S93.1, S93.3, S73.1, S76.-, S83.4, S83.5, S83.6, S86.-, S93.4, S93.5, S93.6, S96.-, T03.3, T13.2, T13.5, S74.-, S75.-, S77.-, S84.-, S85.-, S87.-, S94.-, S95.-, S97.-, T04.3, T13.3, T13.4<br>- Category 4 (underlying diseases/risk factors (e.g., dementia, Parkinson’s disease, visual impairment, dizziness)): R26.0, R26.2, R26.8, R29.6, R54<br>- <u>Category 5:</u> age ≥ 65 years

Cognitive impairment or dementia	F00, F01, F02, F03, F04, F067, F107, G310, G311, G318, R41
Stroke	Ischemic stroke: I63, I64<br>Hemorrhagic stroke: I60, I61
Delirium	F050, F051, F058, F059, F104, F114, F124, F134, F144, F154, F164, F174, F184, F194
GI-bleeding	I850, K228, K250, K252, K254, K256, K260, K262, K264, K266, K270, K272, K274, K276, K280, K282, K284, K286, K290, K625, K920, K921, K922
Peptic ulcer disease	K221, K223, K251, K253, K255, K257, K259, K261, K263, K265, K267, K269, K271, K273, K275, K277, K279, K281, K283, K285, K287, K289, K631, K633
nonGI Bleeding	D500, D62x, D683, D698, D699, G951, I230, I312, I600, I601, I602, I603, I604, I605, I606, I607, I608, I609, I610, I611, I612, I613, I614, I615, I616, I618, I619, I620, I621, I629, I690, I691, I692, J942, K661, M250, N020, N021, N022, N023, N024, N025, N026, N027, N028, N029, N421, N938, N939, R040, R041, R042, R048, R049, R31x, R58x, S064

Table S3: Specification of exposure to comedication

Drug group	Indicators	BNF codes
**Antidepressants**	All	
Selective serotonin reuptake inhibitors (SSRIs)		Citalopram hydrobromide (0403030D0), Citalopram hydrochloride (0403030Z0), Escitalopram (0403030X0) Fluoxetine hydrochloride (0403030E0), Fluvoxamine maleate (0403030L0), Paroxetine hydrochloride (0403030P0), Sertraline hydrochloride (0403030Q0)
Serotonin–norepinephrine reuptake inhibitor (SNRIs)		Venlafaxine (0403040W0), Duloxetine hydrochloride (0403040Y0), Duloxetine hydrochloride (0704020AA)
Tricyclic antidepressants (TCAs)		Amitriptyline hydrochloride (0403010B0), Amoxapine (0403010C0), Clomipramine hydrochloride (0403010F0) Dosulepin hydrochloride (0403010J0), Doxepin (0403010L0), Imipramine hydrochloride (0403010N0) Lofepramine hydrochloride (0403010R0), Nortriptyline (0403010V0), Trimipramine maleate (0403010Y0)
NASSAs noradrenergic and specific serotonergic antidepressants / tetracyclic Antidepressant (NASSAs)		Maprotiline hydrochloride (0403010S0), Mianserin hydrochloride (0403010T0), Mirtazapine (0403040X0)
Monoamine oxidase inhibitors (MAOIs)		Isocarboxazid (0403020H0), Moclobemide (0403020K0) Phenelzine sulfate (0403020M0), Tranylcypromine sulfate (0403020Q0)
Other ADs		Trazodone hydrochloride (0403010X0), Agomelatine (0403040Z0), Nefazodone hydrochloride (0403040T0), Reboxetine (0403040U0), Vortioxetine (0403040AB), Bupropion (0410020A0)
**Credible meds:**	C5	
**Known risk of TdP**		Amiodarone hydrochloride (0203020D0), Anagrelide hydrochloride (0901040A0), Azithromycin (0501050A0), Chloroquine phosphate (0504010F0), Chloroquine sulfate (0504010G0), Chloroquine phosphate with proguanil hydrochloride (0504010Z0), Chlorpromazine hydrochloride (0402010D0), Chlorprothixene (0402010F0), Cilostazol (0206040X0), Ciprofloxacin (0501120L0), Clarithromycin (0501050B0), Disopyramide (0203020F0), Disopyramide phosphate (0203020G0), Domperidone (0406000J0), Donepezil hydrochloride (0411000D0), Dronedarone hydrochloride (0203020X0), Erythromycin (0501050C0), Erythromycin ethylsuccinate (0501050H0), Erythromycin stearate (0501050N0), Flecainide acetate (0203020I0), Fluconazole (0502010B0), Haloperidol (0402010J0), Haloperidol decanoate (0402020T0) Hydroxychloroquine sulfate (1001030C0), Levofloxacin (0501120X0), Levomepromazine maleate (0402010K0), Levomepromazine hydrochloride (0402010L0), Methadone hydrochloride (0309010S0), Methadone hydrochloride (0407020M0), Methadone hydrochloride (0410030C0), Moxifloxacin (0501120Y0), Ondansetron

	hydrochloride (0406000S0), Papaverine hydrochloride (0704050J0), Pentamidine isetionate (0504080P0), Pimozide (0402010R0), Procainamide hydrochloride (0203020S0), Propofol (1501010L0), Quinidine sulfate (0203020U0), Sotalol hydrochloride (0204000T0), Sulpiride (0402010U0), Terfenadine (0304010X0) Terlipressin (0605020T0), Thioridazine (0402010W0) Vandetanib (0801050BG)
**Possible risk of TdP**	Alfuzosin hydrochloride (0704010A0), Alimemazine tartrate (0304010Y0), Apalutamide (0803042V0), Apomorphine hydrochloride (0409010A0), Apomorphine hydrochloride hemihydrate (0409010AC) Aripiprazole (0402010AD), Aripiprazole (0402020AD) Artemether with lumefantrine (0504010AA), Asenapine (0402030R0), Atomoxetine hydrochloride (0404000S0) Bedaquiline (0501090V0), Bicalutamide (0803042A0) Bosutinib (0801050BL), Buprenorphine (0407020B0), Buprenorphine hydrochloride (0410030A0), Buprenorph hydrochloride/naloxone hydrochloride (0410030B0), Cabozantinib (0801050BR), Capecitabine (0801030V0), Carbetocin (0701010W0), Clozapine (0402010C0), Dabrafenib (old) (0802040AI), Dasatinib (0801050AN), Degarelix (0803042R0), Dextromethorphan hydrobromide (0309010L0), Efavirenz/emtricitabine/tenofovir disoproxil (0503010AN), Efavirenz (0503010D0), Encorafenib (0801050CM), Retigabine (0408010AJ), Felbamate (0408010AA), Fingolimod (0802040AD), Fluorouracil (Antimetabolites) (0801030H0), Flupentixol hydrochloride (0402010H0), Flupentixol decanoate (0402020G0), Flupentixol hydrochloride (0403040F0) Granisetron (0406000AF), Granisetron hydrochloride (0406000M0), Imatinib mesilate (0801050AA), Isradipine (0206020I0), Ketanserin (0205080A0), Lacidipine (0206020K0), Lapatinib (0801050AT), Leuprorelin acetate (0803042N0), Levetiracetam (0408010A0), Lithium carbonate (0402030K0), Lithium citrate (0402030P0), Lofexidine hydrochloride (0410030D0), Lopinavir and ritonavir (0503010G0), Lurasidone (0402010AI), Melperone hydrochloride (0402010AF), Midostaurin (0801050CK), Mifepristone/misoprostol (0701021A0), Mirabegron (0704020AE), Nicardipine hydrochloride (0206020Q0) Nilotinib (0801050AS), Norfloxacin (0501120Q0), Ofloxacin (0501120P0), Osimertinib (0801050CG), Oxytocin (0701010T0), Paliperidone (0402010AE), Paliperidone (0402020AB), Palonosetron hydrochloride (0406000AB), Netupitant/palonosetron hydrochloride (0406000AG), Pasireotide (0803043Q0), Pazopanib

	(0801050AZ), Perphenazine (0402010Q0), Pitolisant hydrochloride (0404000W0), Primaquine (0504010K0), Promethazine hydrochloride (0304010W0), Promethazine teoclate (0406000V0), Relugolix (0803042X0), Ribociclib (0801050CR), Rilpivirine (0503010AG), Dolutegravir/rilpivirine (0503010AP) Saquinavir mesylate (0503010R0), Siponimod (0802040BB), Sorafenib (0801050AJ), Sunitinib (0801050AM), Tacrolimus (0802020T0), Tamoxifen citrate (0803041S0), Telithromycin (0501050T0), Tetrabenazine (0409030C0), Tizanidine hydrochloride (1002020T0), Tolterodine (0704020N0), Toremifene citrate (0803041U0), Tramadol hydrochloride (040702040), Vardenafil (0704050AA), Zotepine (0402010AC), Zuclopenthixol acetate (040201010), Zuclopenthixol hydrochloride (0402010T0), Zuclopenthixol decanoate (0402020Z0), Mirtazapine (0403040X0), Venlafaxine (0403040W0)
**Conditional risk of TdP**	Abiraterone (0803042T0), Amantadine hydrochloride (0409010B0), Amantadine hydrochloride (0503040C0) Amisulpride (0402010A0), Amphotericin (0502030A0) Atazanavir and cobicistat (0503010AL), Atazanavir (0503010L0), Bendroflumethiazide (0202010B0), Bendroflumethiazide/potassium (0202080B0), Chloral hydrate (0401010B0), Cimetidine (0103010D0), Clofazimine (0501100C0), Diltiazem hydrochloride (0206020C0), Diphenhydramine hydrochloride (0304010N0), Diphenhydramine hydrochloride (030902040), Diphenhydramine hydrochloride/pholcodine (0309020AB), Esomeprazole (0103050E0), Famotidine (0103010H0), Furosemide (0202020L0), Furosemide/potassium (0202080K0) Galantamine (0411000F0), Hydrochlorothiazide (0202010L0), Co-amilozide (Amiloride hydrochloride/hydrochlorothiazide) (0202040C0), Co-triamterzide(Triamterene/hydrochlorothiazide) (0202040H0), Co-zidocapt (Hydrochlorothiazide/captopril) (0205051G0) Olmesartan medoxomil /amlodipine/hydrochlorothiazide (0205052AC), Olmesartan medoxomil/hydrochlorothiazide (0205052Y0), Hydroxyzine hydrochloride (0304010J0) Indapamide (0202010P0), Perindopril tosilate /indapamide (0205051AB), Itraconazole (0502010C0) Ivabradine (0206030Y0), Ketoconazole (0502020A0) Lansoprazole (0103050L0), Loperamide hydrochloride (0104020L0), Loperamide hydrochloride and simeticone (0104020P0), Metoclopramide hydrochloride (0406000P0), Metolazone (0202010V0), Metronidazole (0501110C0), Nelfinavir mesilate (0503010C0), Olanzapine (040201060), Olanzapine embonate

		(0402020AC), Omeprazole (0103050P0), Pantoprazole (0103050R0), Piperacillin sodium/tazobactam sodium (0501014S0), Posaconazole (0502010A0), Propafenone hydrochloride (0203020R0), Quetiapine (0402010AB) Quinine sulfate (0504010Y0), Ranolazine (0206030Z0) Risperidone (040201030), Risperidone (0402020AA) Solifenacin/tamsulosin (0704010W0), Solifenacin (0704020AB), Solifenacin/Tamsulosin (0704020AF) Telaprevir (0503030F0), Telaprevir (0503032B0) Torasemide (0202020U0), Voriconazole (0502010D0) Ziprasidone hydrochloride (0402010AG), Fluoxetine hydrochloride (0403030E0), Fluvoxamine maleate (0403030L0), Paroxetine hydrochloride (0403030P0) Sertraline hydrochloride (0403030Q0), Trazodone hydrochloride (0403010X0)
**Renin-angiotensin system (RAAS) drugs** (ACE-Inhibitor/ Angiotensin Receptor Blocker/Aliskiren)	C7, OH1, OH2	Aliskiren (0205053A0), Azilsartan medoxomil (0205052AD), Candesartan cilexetil (0205052C0) Captopril (0205051F0), Cilazapril (0205051E0) Co-zidocapt (Hydrochlorothiazide/captopril) (0205051G0), Enalapril maleate (0205051I0) Enalapril maleate with diuretic (0205051H0) Eprosartan (0205052W0), Fosinopril sodium (0205051J0), Imidapril hydrochloride (0205051W0) Irbesartan (0205052I0), Irbesartan with diuretic (0205052A0), Lisinopril (0205051L0), Lisinopril with diuretic (0205051K0), Losartan potassium (0205052N0), Losartan potassium with diuretic (0205052P0), Moexipril hydrochloride (0205051C0), Olmesartan medoxomil (0205052B0), Olmesartan medoxomil/amlodipine (0205052AB), Olmesartan medoxomil/amlodipine/hydrochlorothiazide (0205052AC), Olmesartan medoxomil/hydrochlorothiazide (0205052Y0), Perindopril arginine (0205051Y0), Perindopril arginine with diuretic (0205051Z0), Perindopril erbumine (0205051M0), Perindopril erbumine with diuretic (0205051N0), Perindopril tosilate (0205051AA), Perindopril tosilate/indapamide (0205051AB), Perindopril with calcium channel blocker (0205051AC), Quinapril hydrochloride (0205051Q0), Quinapril hydrochloride with diuretic (0205051P0), Ramipril (0205051R0), Ramipril with calcium channel blocker (0205051S0), Sacubitril/valsartan (0205052AE), Telmisartan (0205052Q0), Telmisartan with diuretic (0205052R0), Trandolapril (0205051U0), Trandolapril with calcium channel blocker (0205051V0), Valsartan (0205052V0), Valsartan with diuretic (0205052X0)
**Calcium Channel Blocker**	C7, OH1, OH2, CO1	Amlodipine (0206020A0), Diltiazem hydrochloride (0206020C0), Felodipine (0206020F0), Isradipine (0206020I0), Lacidipine (0206020K0), Lercanidipine hydrochloride (0206020L0), Nicardipine hydrochloride

		(0206020Q0), Nifedipine (0206020R0), Nimodipine (0206020M0), Nisoldipine (0206020W0), Trimetazidine hydrochloride (0206020B0), Valsartan/amlodipine (0206020Z0), Verapamil hydrochloride (0206020T0)
**Diuretics:**	C7, OH1, OH2, D1, D2, F1, F4	
Thiazides and related diuretics		Bendroflumethiazide (0202010B0), Chlorothiazide (0202010D0), Chlortalidone (0202010F0), Cyclopenthiazide (0202010J0), Hydrochlorothiazide (0202010L0), Indapamide (0202010P0), Metolazone (0202010V0), Polythiazide (0202010X0), Xipamide (0202010Y0)
Loop diuretics		Bumetanide (0202020D0), Furosemide (0202020L0) Torasemide (0202020U0)
Potassium-sparing diuretics and aldosterone antagonists		Amiloride hydrochloride (0202030C0), Eplerenone (0202030X0), Finerenone (0202030Y0), Spironolactone (0202030S0), Triamterene (0202030W0)
Potassium sparing diuretics and compounds		Amiloride hydrochloride with loop diuretics (0202040D0), Amiloride hydrochloride with thiazides (0202040A0), Co-amilofruse (Amiloride hydrochloride/frusemide) (0202040B0), Co-amilozide (Amiloride hydrochloride/hydrochlorothiazide) (0202040C0), Co-flumactone (Hydroflumethiazide/spironolactone) (0202040G0) Co-triamterzide(Triamterene/hydrochlorothiazide) (0202040H0), Spironolactone with loop diuretics (0202040T0), Spironolactone with thiazides (0202040S0), Triamterene with loop diuretics (0202040U0), Triamterene with thiazides (0202040V0)
Diuretics with potassium		Bendroflumethiazide/potassium (0202080B0), Bumetanide/Amiloride Hydrochloride (0202080D0), Bumetanide/potassium (0202080C0), Furosemide/potassium (0202080K0)
**Beta-adrenoceptor blocking drugs**	C7, OH1, OH2	Acebutolol hydrochloride (0204000C0), Atenolol (0204000E0), Atenolol with calcium channel blocker (0204000U0), Atenolol with diuretic (0204000F0), Bisoprolol fumarate (0204000H0), Bisoprolol fumarate/aspirin (0204000AC), Carvedilol (020400080) Celiprolol hydrochloride (020400060), Co-prenozide (Oxprenolol hydrochloride/cyclopenthiazide) (0204000Y0), Co-tenidone (Atenolol/chlortalidone) (020400040), Labetalol hydrochloride (0204000I0), Metoprolol tartrate (0204000K0), Metoprolol tartrate with diuretic (0204000W0), Nadolol (0204000M0), Nebivolol (0204000AB), Oxprenolol hydrochloride (0204000N0), Pindolol (0204000P0), Pindolol with diuretic (020400010), Propranolol hydrochloride (0204000R0), Propranolol hydrochloride with diuretic (0204000Q0), Sotalol hydrochloride (0204000T0), Timolol (0204000V0), Timolol with diuretic (020400030)

**Alpha blocker, antihypertensives**	C7, OH1, OH2, F1, F4	Doxazosin (0205040D0), Indoramin (0205040I0), Phenoxybenzamine (0205040M0), Phentolamine (0205040P0), Prazosin (0205040S0), Terazosin (0205040V0)
**Centrally acting antihypertensives**	C7, OH1, OH2, F1, F4	Clonidine hydrochloride (0205020E0), Clonidine hydrochloride (0407042F0), Guanfacine hydrochloride (0205020G0), Guanfacine (0404000V0), Methyldopa (0205020H0), Moxonidine (0205020M0)
**Nitrates**	OH1, OH2, F1, F4	Amyl nitrite (0206010A0), Glyceryl trinitrate (0206010F0), Isosorbide dinitrate (0206010I0), Isosorbide mononitrate (0206010K0)
**Antipsychotic drugs**		
	OH1, OH2, F1, F4, S1, S2	Paliperidone (0402010AE 0402020AB), Ziprasidone (0402010AG), Risperidone (040201030 0402020AA), Amisulpride (0402010A0), Sulpiride (0402010U0), Clozapine (0402010C0), Olanzapine (040201060 0402020AC), Quetiapine (0402010AB), Asenapine (0402030R0), Zotepine (0402010AC), Aripiprazole (0402010AD), Aripiprazole (0402020AD), Cariprazine (0402010AJ), Lurasidone (0402010AI) Chlorpromazine (0402010D0), Fluphenazine (0402010I0 0402020L0), Perphenazine (0402010Q0), Thioridazine (0402010W0), Pipotiazine (0402020V0), Haloperidol (0402020T0 0402010J0), Benperidol (0402010B0), Melperone (0402010AF), Pimozide (0402010R0), Flupentixol (0402020G0 0402010H0), Chlorprothixene (0402010F0), Zuclopenthixol (040201010 0402010T0 0402020Z0), Loxapine (0402010M0), Levomepromazine (0402010L0, 0402010K0), Pericyazine (0402010P0), Promazine hydrochloride (0402010S0), Trifluoperazine (0402010X0)
Atypical		Paliperidone (0402010AE 0402020AB), Ziprasidone (0402010AG), Risperidone (040201030 0402020AA), Amisulpride (0402010A0), Sulpiride (0402010U0), Clozapine (0402010C0), Olanzapine (040201060 0402020AC), Quetiapine (0402010AB), Asenapine (0402030R0), Zotepine (0402010AC), Aripiprazole (0402010AD), Aripiprazole (0402020AD), Cariprazine (0402010AJ), Lurasidone (0402010AI)
**Antihistamines**	F1, F4	Acrivastine (0304010A0), Alimemazine tartrate (0304010Y0), Azatadine maleate (0304010C0), Bilastine (0304010AF), Brompheniramine maleate (0304010F0), Cetirizine hydrochloride (0304010I0), Chlorphenamine maleate (0304010G0), Clemastine fumarate (0304010H0), Cyproheptadine hydrochloride (0304010K0), Desloratadine (0304010AB), Diphenhydramine hydrochloride (0304010N0), Fexofenadine hydrochloride (0304010E0), Hydroxyzine hydrochloride (0304010J0), Ketotifen fumarate (0304010AG), Levocetirizine (0304010AC), Loratadine (0304010D0), Mizolastine (0304010AA), Promethazine

		hydrochloride (0304010W0), Rupatadine fumarate (0304010AE), Terfenadine (0304010X0), Promethazine teoclate (0406000V0), Doxylamine succinate/pyridoxine hydrochloride (0406000AA), Dimenhydrinate/cinnarizine (0406000AC), Meclozine hydrochloride (0406000N0)
**Benzodiazepines**	F1, F4, D1, D2	Flurazepam hydrochloride (0401010L0), Loprazolam mesilate (0401010N0), Lormetazepam (0401010P0), Midazolam Maleate (0401010Q0), Nitrazepam (0401010R0), Temazepam (0401010T0), Alprazolam (0401020A0), Bromazepam (0401020G0), Diazepam (0401020K0), Lorazepam (0401020P0), Oxazepam (0401020T0), Chlordiazepoxide hydrochloride (0401020E0)
**Z-Drugs**	F1, F4, D1, D2	Zaleplon (0401010W0), Zolpidem tartrate (0401010Y0) Zopiclone (0401010Z0)
**GABA-Agonists** (+Benzodiazepines, z-Drugs)	D1, D2	Amobarbital (0401030C0), Amobarbital sodium (0401030E0), Butobarbital (0401030H0), Secobarbital sodium (0401030T0), Phenobarbital (0408010N0) Phenobarbital sodium (0408010P0), Clobazam (040801060), Clonazepam (0408010F0, 0408020D0), Midazolam maleate (0408020V0), Midazolam hydrochloride (0408020W0), Midazolam hydrochloride (1501041T0), Baclofen (1002020C0)
**Overactive bladder and urge incontinence medications**	F1, F4	Darifenacin hydrobromide (0704020AC), Fesoterodine fumarate (0704020AD), Flavoxate hydrochloride (0704020G0)<br>Mirabegron (0704020AE), Oxybutynin (0704020J0), Propiverine hydrochloride (0704020P0), Solifenacin (0704020AB), Solifenacin/Tamsulosin (0704020AF), Tolterodine (0704020N0), Trospium chloride (0704020Z0)
**Opioids** (without morphine)	F1, F4, D1, D2, CO1	Buprenorphine (0407020B0), Codeine phosphate (0407020C0), Dextromoramide tartrate (0407020D0) Dextropropoxyphene (0407020E0), Diamorphine hydrochloride (Systemic) (0407020K0), Dihydrocodeine tartrate (0407020G0), Dipipanone hydrochloride (0407020H0), Fentanyl (0407020A0), Hydromorphone hydrochloride (040702050), Meptazinol hydrochloride (0407020L0), Methadone hydrochloride (0407020M0), Nalbuphine hydrochloride (0407020Y0), Oxycodone (0407020Z0), Oxycodone hydrochloride (0407020AD), Oxycodone hydrochloride/naloxone hydrochloride (0407020AF), Papaveretum (0407020AB), Pentazocine hydrochloride (0407020T0), Pentazocine lactate (0407020U0), Pethidine hydrochloride (0407020V0), Tapentadol hydrochloride (0407020AG), Tapentadol phosphate (0407020AH), Tramadol hydrochloride (040702040)
**Antiepileptic drugs**	F1, F4	Brivaracetam (0408010AL), Cannabidiol (0408010AM), Carbamazepine (0408010C0), Cenobamate

		(0408010AN), Clobazam (040801060), Clonazepam (0408010F0), Eslicarbazepine acetate (0408010AI), Ethosuximide (0408010I0), Felbamate (0408010AA), Gabapentin (0408010G0), Lacosamide (0408010AH), Lamotrigine (0408010H0), Levetiracetam (0408010A0), Mesuximide (0408010K0), Oxcarbazepine (0408010D0), Perampanel (0408010AK), Phenobarbital (0408010N0), Phenobarbital sodium (0408010P0), Phenytoin (0408010Z0), Phenytoin sodium (0408010Q0), Pregabalin (0408010AE), Primidone (0408010U0), Retigabine (0408010AJ), Rufinamide (0408010AF), Semisodium Valproate (0408010AC), Sodium valproate (0408010W0), Stiripentol (0408010AG), Sultiame (0408010Y0), Tiagabine (0408010AB), Topiramate (040801050), Valproic acid (040801020), Vigabatrin (0408010X0), Zonisamide (0408010AD), Clonazepam (0408020D0), Midazolam hydrochloride (0408020W0), Midazolam maleate (0408020V0), Midazolam hydrochloride (1501041T0), Phenytoin sodium (0408020T0), Valproic acid (0402030Q0), Gabapentin (Neuropathic Pain) (0407030AD)
**Vasodilators in cardiac disease**	F1, F4	Amyl nitrite (0206010A0), Glyceryl trinitrate (0206010F0), Isosorbide dinitrate (0206010I0), Isosorbide mononitrate (0206010K0), Nicorandil (0206030N0), Dipyridamole (0209000L0, 0209000V0), Vericiguat (0205010AC)
**Anticholinergic drugs (ACB score 3*)**	F1, F4, D1, D2, CO1	Amoxapine (0403010C0), Atropine sulfate (0102000AC) Co-phenotrope (Diphenox hydrochloride/atropine sulfate) (0104020H0), Atropine sulfate (1501030D0), Belladonna alkaloids (0102000H0), Benzatropine mesilate (0409020E0), Biperiden hydrochloride (0409020G0), Brompheniramine maleate (0304010F0), Hyoscine butylbromide (0102000N0), Hyoscine (0406000A0), Hyoscine hydrobromide (0406000L0), Hyoscine hydrobromide (1501030H0), Carisoprodol (1002020G0), Chlorphenamine maleate (0304010G0), Chlorpromazine hydrochloride (0402010D0), Clemastine fumarate (0304010H0), Dicycloverine hydrochloride (0102000J0), Dicycloverine hydrochloride compound preparations (0102000K0), Dimenhydrinate/cinnarizine (0406000AC), Diphenhydramine hydrochloride (0304010N0), Diphenhydramine hydrochloride (030902040), Diphenhydramine hydrochloride/pholcodine (0309020AB), Ammonium chloride/diphenhydramine hydrochloride (0309020AH), Hydroxyzine hydrochloride (0304010J0), Meclozine hydrochloride (0406000N0), Orphenadrine hydrochloride (0409020N0), Oxybutynin (0704020J0), Oxybutynin hydrochloride (0704040G0), Procyclidine hydrochloride (0409020S0), Propantheline

		bromide (0102000Y0), Thioridazine (0402010W0), Trifluoperazine (0402010X0), Trihexyphenidyl hydrochloride (0409020C0)
**Anticholinergic drugs (ACB score 2*)**	F1, F4, D1, D2, CO1	Amantadine hydrochloride (0409010B0), Amantadine hydrochloride (0503040C0), Chlorprothixene (0402010F0), Cimetidine (0103010D0), Clozapine (0402010C0), Cyproheptadine hydrochloride (0304010K0), Darifenacin hydrobromide (0704020AC), Fesoterodine fumarate (0704020AD), Flavoxate hydrochloride (0704020G0), Fluphenazine hydrochloride (0402010I0), Fluphenazine decanoate (0402020L0), Levomepromazine maleate (0402010K0), Levomepromazine hydrochloride (0402010L0), Loxapine succinate (0402010M0), Nefopam hydrochloride (0407010P0), Olanzapine (040201060), Olanzapine embonate (0402020AC), Oxcarbazepine (0408010D0), Pericyazine (0402010P0), Perphenazine (0402010Q0), Pethidine hydrochloride (0407020V0), Pimozide (0402010R0), Pregabalin (0408010AE), Pridinol mesilate (1002020Z0), Promazine hydrochloride (0402010S0), Promethazine hydrochloride (0304010W0), Promethazine teoclate (0406000V0), Propiverine hydrochloride (0704020P0), Dextropropoxyphene (0407020E0), Secobarbital sodium (0401030T0), Solifenacin/tamsulosin (0704010W0), Solifenacin (0704020AB), Solifenacin/Tamsulosin (0704020AF), Tizanidine hydrochloride (1002020T0), Tolterodine (0704020N0), Trospium chloride (0704020Z0), Zotepine (0402010AC), Pseudoephedrine hydrochloride/dextromethorphan hydrobromide (0309020AF), Naltrexone/Bupropion (0405010T0), Naltrexone/bupropion (0405020U0)
**Alpha blocker, prostate hyperplasia**	F1, F4, VD1	Alfuzosin (0704010A0), Indoramin (0704010M0), Solifenacin/tamsulosin (0704010W0), Tamsulosin and dutasteride (0704010V0), Tamsulosin (0704010U0) Terazosin (0704010T0)
**Drugs for dementia**	F3, CD1	Donepezil hydrochloride (0411000D0), Galantamine (0411000F0), Idebenone (0411000H0), Memantine hydrochloride (0411000G0), Rivastigmine (0411000E0)
**Glucocorticoids**	D1, D2	Beclometasone dipropionate (Systemic) (0105020G0), Budesonide (0105020A0), Hydrocortisone (0105020C0) Hydrocortisone acetate (0105020B0), Prednisolone (0105020F0), Prednisolone sodium metasulphobenzoate (0105020D0), Prednisolone sodium phosphate (0105020E0), Betamethasone (0603020B0), Betamethasone sodium phosphate (0603020C0), Cortisone acetate (0603020F0), Deflazacort (0603020I0), Dexamethasone (0603020G0), Dexamethasone phosphate (0603020AA), Dexamethasone sodium phosphate (0603020H0),

		Hydrocortisone (0603020J0), Hydrocortisone sodium phosphate (0603020L0), Hydrocortisone sodium succinate (0603020M0), Methylprednisolone (0603020S0), Methylprednisolone aceponate (0603020AC), Methylprednisolone sodium succinate (0603020K0), Prednisolone (0603020T0), Prednisone (0603020X0), Triamcinolone acetonide (0603020Z0)
**Serotonergic drugs**	S1, S2	Selegiline hydrochloride (0409010T0), Rasagiline mesilate (0409010Y0), Tramadol hydrochloride (040702040), Methadone hydrochloride (0309010S0), Methadone hydrochloride (0407020M0), Methadone hydrochloride (0410030C0), Fentanyl (0407020A0) (1501043F0), Metoclopramide hydrochloride (0406000P0), Granisetron (0406000AF), Granisetron hydrochloride (0406000M0), Ondansetron hydrochloride (0406000S0), Carbamazepine (0408010C0), Oxcarbazepine (0408010D0), Eslicarbazepine acetate (0408010AI), Valproic acid (0402030Q0), Valproic acid (040801020), Semisodium Valproate (0408010AC), Sodium valproate (0408010W0), Buspirone hydrochloride (0401020B0), Lithium carbonate (0402030K0), Lithium citrate (0402030P0), Triptans, Atypical antipsychotics, mirtazapine, isocarboxazid, moclobemide, phenelzine
**Triptans**	S1, S2	Almotriptan (0407041B0), Eletriptan (0407041AA), Frovatriptan (0407041AB), Naratriptan hydrochloride (0407041M0), Rizatriptan (0407041R0), Sumatriptan succinate (0407041T0), Zolmitriptan (0407041Z0)
**Drugs known to increase the risk of hyponatremia**	H1	Carbamazepine (0408010C0), Oxcarbazepine (0408010D0), Eslicarbazepine acetate (0408010AI), Desmopressin acetate (0605020E0), Terlipressin (0605020T0), Semisodium Valproate (0408010AC), Sodium valproate (0408010W0), Valproic acid (0402030Q0), Valproic acid (040801020), Proton pump inhibitors, Thiazides and related diuretics, Loop diuretics, Atypical antipsychotics, Tricyclic antidepressants, Maprotiline hydrochloride (0403010S0), Mianserin hydrochloride (0403010T0), Mirtazapine (0403040X0)
**Antiplatelets**	G1, G2, G3, B1, B2	Aspirin (0209000A0), Aspirin/bisoprolol (0204000AC), Clopidogrel (0209000C0), Dipyridamole (0209000L0), Dipyridamole and aspirin (0209000V0), Eptifibatide (0209000X0), Prasugrel (0209000Y0), Ticagrelor (0209000Z0), Ticlopidine hydrochloride (0209000U0), Cilostazol (0206040X0), Iloprost (0205010V0), Epoprostenol (0208010E0)
**Anticoagulants**	G1, G2, G3, B1, B2	Bemiparin sodium (0208010AC), Dalteparin sodium (0208010L0), Danaparoid sodium (0208010C0), Enoxaparin (0208010D0), Fondaparinux sodium (0208010AB), Heparin calcium (0208010N0), Heparin flushes (0208010P0), Heparin sodium (0208010K0),

		Tinzaparin sodium (0208010W0), Acenocoumarol (0208020H0), Apixaban (0208020Z0), Dabigatran etexilate (0208020X0), Edoxaban (0208020AA), Pentosan polysulfate sodium (0208020I0), Phenindione (0208020N0), Phenprocoumon (0208020S0), Rivaroxaban (0208020Y0), Warfarin sodium (0208020V0)
**NSAIDs (without coxibs)**	G1, G2, G3	Aceclofenac (100101080), Acemetacin (100101050), Azapropazone (1001010B0), Benorilate (1001010H0), Dexibuprofen (1001010AM), Dexketoprofen (1001010AE), Diclofenac potassium (1001010AG), Diclofenac sodium (1001010C0), Diflunisal (1001010D0), Etodolac (1001010E0), Fenbufen (1001010F0), Fenoprofen (1001010G0), Flurbiprofen (1001010I0), Ibuprofen (1001010J0), Ibuprofen lysine (1001010AD), Ibuprofen sodium dihydrate (1001010AP), Indometacin (1001010K0), Ketoprofen (1001010L0), Lornoxicam (1001010AI), Mefenamic acid (1001010N0), Meloxicam (1001010AA), Nabumetone (1001010X0), Naproxen (1001010P0), Naproxen sodium (100101070), Phenylbutazone (1001010Q0), Piroxicam (1001010R0), Sulindac (1001010S0), Tenoxicam (100101040), Tiaprofenic acid (1001010T0), Ibuprofen/pseudoephedrine hydrochloride (0309020AA), Paracetamol and ibuprofen (0407010AD), Aspirin (0407010B0), Aspirin and caffeine (0407010AA), Aspirin and papaveretum (0407010A0), Aspirin combined preparations (0407010W0), Co-codaprin (Codeine phosphate/aspirin) (0407010M0)
**Proton pump inhibitors**	G1, G2, H1	Esomeprazole (0103050E0), Helicobacter pylori eradication therapy (0103050A0), Lansoprazole (0103050L0), Omeprazole (0103050P0), Pantoprazole (0103050R0), Rabeprazole sodium (0103050T0)
**Drugs for urinary retention (5α-Reductase-Inhibitor)**	VD1	Finasteride (0604020C0), Dutasteride (0604020B0)
**Hypnotics**	I1	Zaleplon (0401010W0), Zolpidem tartrate (0401010Y0), Zopiclone (0401010Z0), Oxazepam (0401020T0), Lorazepam (0401020P0), Bromazepam (0401020G0), Alprazolam (0401020A0), Lormetazepam (0401010P0), Temazepam (0401010T0), Chlorpromazine (0402010D0), Diphenhydramine hydrochloride (0304010N0), Hydroxyzine hydrochloride (0304010J0), Promethazine hydrochloride (0304010W0), Melatonin (0401010AD), Mirtazapine (0403040X0) (15mg), Amitriptyline hydrochloride (0403010B0) (10, 25 or 30mg), Doxepin (0403010L0) (10, 25 or 30mg), Imipramine hydrochloride (0403010N0) (10, 25 or 30mg), Trimipramine maleate (0403010Y0) (10, 25 or 30mg)
**Drugs for erectile dysfunction**	SD1	Alprostadil (0704050B0), Avanafil (0704050AC), Papaverine hydrochloride (0704050J0),

	Phentolamine/aviptadil (0704050AD), Sildenafil (Erectile Dysfunction) (0704050Z0), Tadalafil (0704050R0), Vardenafil (0704050AA), Yohimbine hydrochloride (0704050Y0)

*As the modified Anticholinergic Risk Scale (mARS) was published ten years ago, we conducted a structured literature search to identify other international scores that assess the anticholinergic burden (ACB) of drugs on a scale of 0 to 3. Here we found six additional scores from Brazil, Germany, Korea, USA, Spain and Sweden. The mean value was then calculated for each of the drugs mentioned, whereby the value was always rounded down regardless of the decimal place. We thus identified a total of 146 drugs registered in Scotland (26 with ACB 3, 37 with ACB 2 and 83 with ACB 1).
