# Supplementary material for: Potential deprescribing indications for antidepressants between 2012 and 2019: repeated cross-sectional analysis in two Scottish health boards
Source: BMC Med. 2024 Sep 11;22:378. doi: 10.1186/s12916-024-03584-9 (PMC11389559; doi:10.1186/s12916-024-03584-9)
Supplement: Supplementary file 3 — Additional file 3: Tables S4-S9. Table S4. Distribution of antidepressant groups among all antidepressant users. Table S5. Prevalence of each potential high-risk use indicator in 2012 and 2019. Table S6. Proportion of antidepressant users triggering indicators targeting specific adverse drug reaction risks (2012). Table S7. Proportion of antidepressant users triggering indicators targeting specific adverse drug reaction risks (2019). Table S8. PDIs among antidepressant users in 2012 and 2019. Table S9. Sensitivity analysis. [file 12916_2024_3584_MOESM3_ESM.pdf]

Additional file 3:

Table S4: Distribution of antidepressant groups among all antidepressant users

Table S5: Prevalence of each potential high-risk use indicator in 2012 and 2019

Table S6: Proportion of antidepressant users triggering indicators targeting specific adverse drug reaction risks (2012) and their distribution among age groups

Table S7: Proportion of antidepressant users triggering indicators targeting specific adverse drug reaction risks (2019) and their distribution among age groups

Table S8: Potential deprescribing indications (PDIs) (i.e., simultaneous long-term and potential high-risk use) in 2012 and 2019

Table S9: Sensitivity analysis for long-term and potential high-risk antidepressant use in 2012 and 2019

Table S4: Distribution of antidepressant groups among all antidepressant users

	**Second quarter 2012**	**Second quarter 2019**		
	No. of patients (crude %)	No. of patients (crude %, **age-sex standardised[a] %)**	**Relative risk 2019 vs 2012** (95% CI)	
			Crude	Age-sex stand.
**Type of antidepressant drug class**				
SSRI	39,791/73,600 (54.1)	51,244/92,601 (55.3; **54.9**)	1.02 (1.01-1.03)	1.02 (1.01-1.03)
TCA	25,198/73,600 (34.2)	25,833/92,601 (27.9; **28.4**)	0.81 (0.80-0.83)	0.83 (0.82-0.84)
SNRI	5,092/73,600 (6.9)	9,470/92,601 (10.2; **10.3**)	1.48 (1.43-1.53)	1.49 (1.44-1.54)
NASSA	6,865/73,600 (9.3)	13,279/92,601 (14.3; **14.3**)	1.54 (1.50-1.58)	1.53 (1.49-1.58)
MAOI	160/73,600 (0.2)	128/92,601 (0.1; **0.1**)	0.64 (0.50-0.80)	0.64 (0.50-0.82)
Others	2,586/73,600 (3.5)	2,700/92,601 (2.9; **3.0**)	0.83 (0.79-0.88)	0.84 (0.79-0.89)

[a]direct age-sex standardisation to the 2012 population

Table S5: Prevalence of each potential high-risk use indicator in 2012 and 2019

**Indicators**	**2012**		**2019**	
**Cardiovascular adverse effects**	No. of patients	Prevalence (N/D) (%)	No. of patients	Prevalence (N/D) (%)
C1. Dispensed SNRI or TCA (dispensed strength ≥50 mg) or tranylcypromine (D)	10657	0.3	13946	0.4
- and the patient has a previous hospital admission for heart failure (N)	31		51	
C2. Dispensed TCA (dispensed strength ≥50 mg) (D)	5690	1.4	4755	0.9
- and the patient has a previous hospital admission for ischemic heart disease (N)	81		45	
C3. Dispensed >20mg citalopram or >10mg escitalopram (D)	4338	11.2	3017	12.4
- and the patient is aged ≥65 years (risk of QTc prolongation) (N)	487		373	
C4. Dispensed citalopram or escitalopram (D)	21599	0.7	16690	0.5
- and the patient has a previous hospital admission for heart failure, myocardial hypertrophy, bradycardia (atrioventricular block) or myocardial infarction (N)	148		76	
C5. Dispensed citalopram, escitalopram, TCA (dispensed strength ≥50 mg), maprotiline, mianserin (D)	27026	55.4	21333	53.1
- and the patient is also dispensed ≥1 further drug with any risk of TdP (N)	14970		11336	
C6. Dispensed fluoxetine, paroxetine or bupropion (D)	14132	4.3	16144	6.7
- and the patient is also dispensed metoprolol or propranolol (risk of bradycardia) (N)	606		1083	
C7. Dispensed SNRI or TCA (dispensed strength ≥50 mg) or bupropion or tranylcypromine (D)	10710	8.2	13959	6.2
- and the patient is also dispensed ≥3 antihypertensive drugs (N)	883		864	
**Total:** patients at risk of cardiovascular adverse effects (D)	45412	35.3	46089	28.2
**Total:** patient triggering cardiovascular adverse effects (N)	16047		13019	
**Orthostatic hypotension (OH)/dizziness**				
OH1. Dispensed TCA (dispensed strength ≥50 mg) or trazodone or tranylcypromine and aged ≥65 years (D)	2721	70.2	2639	66.3
- and the patient is also dispensed ≥1 further drug with known blood pressure lowering effect (N)	1909		1749	
OH2. Dispensed SSRI or SNRI or mirtazapine and aged ≥65 years (D)	12560	42.3	17516	36.4
- and the patient is also dispensed ≥2 further drugs with blood pressure lowering effect (N)	5319		6380	
**Total:** patients at risk of OH/dizziness (D)	14954	47.5	19690	40.4
**Total:** patient triggering OH/dizziness (N)	7098		7949	
**Falls and fall-related injuries**				
F1. Dispensed SSRI, SNRI, TCA (dispensed strength ≥50 mg), mirtazapine or trazodone and aged ≥65 years (D)	14949	78.1	19685	71.3
- and the patient is also dispensed ≥1 further fall-risk-increasing drug (N)	11673		14026	
F2. Dispensed SSRI, SNRI, TCA (dispensed strength ≥50 mg), mirtazapine or trazodone and aged ≥65 years (D)	14949	5.5	19685	6.1
- and the patient has a previous hospital admission for falls (N)	818		1197	
F3. Dispensed SSRI, SNRI, TCA (dispensed strength ≥50 mg), mirtazapine or trazodone and aged ≥65 years (D)	14949	7.9	19685	8.8

- and the patient has a previous hospital admission for dementia or was recently dispensed antidementia drugs (N)	1179		1729	
F4. Dispensed SSRI, SNRI, TCA (dispensed strength ≥50 mg), mirtazapine or trazodone, aged ≥65 years and has a previous hospital admission for stroke (D)	318	81.4	388	76.5
- and the patient is also dispensed ≥1 further fall-risk-increasing drug (N)	259		297	
**Total:** patients at risk of falls (D)	14949	80.8	19685	75.3
**Total:** patient triggering the risk of falls (N)	12075		14823	
**Cognitive decline**				
CD1. Dispensed anticholinergic antidepressant TCA (dispensed strength ≥50 mg), maprotiline or paroxetine (D)	8125	1.1	6414	1.1
- and the patient has a previous hospital admission for dementia or cognitive impairment or was recently dispensed antidementia drugs (N)	89		69	
**Delirium**				
D1. Dispensed anticholinergic antidepressant TCA (dispensed strength ≥50 mg), maprotiline or paroxetine and has a previous hospital admission for delirium (D)	12	58.3	22	68.2
- and the patient is also dispensed ≥1 further drug known to induce delirium (N)	7		15	
D2. Dispensed anticholinergic antidepressant TCA (dispensed strength ≥50 mg), maprotiline or paroxetine and aged ≥65 years (D)	2697	35.8	2316	33.6
- and the patient is also dispensed ≥2 further drugs known to induce delirium (N)	965		778	
**Total:** patients at risk of delirium (D)	2700	35.9	2319	33.8
**Total:** patient triggering the risk of delirium (N)	969		783	
**Serotonin syndrome**				
S1. Dispensed tranylcypromine (D)	10	30.0	8	25.0
- and the patient is also dispensed ≥1 further serotonergic drug (N)	3		2	
S2. Dispensed SSRI or SNRI or TCA (dispensed strength ≥50 mg) (D)	49630	4.0	64071	4.0
- and the patient is also dispensed ≥2 further serotonergic drugs other than tranylcypromine (N)	1975		2590	
**Total:** patients at risk of serotonin syndrome (D)	49639	4.0	64079	4.0
**Total:** patient triggering the risk of serotonin syndrome (N)	1978		2592	
**Gastrointestinal bleeding**				
G1. Dispensed SSRI or SNRI and aged ≥65 years (D)	10567	21.9	13551	14.9
- and the patient is also dispensed a single of the following without GI-protection: antiplatelet, anticoagulant, NSAID (N)	2311		2017	
G2. Dispensed SSRI or SNRI and aged ≥65 years (D)	10567	6.6	13551	2.5
- and the patient is also dispensed ≥2 of the following: antiplatelet, anticoagulant, NSAID (regardless of GI-protection) (N)	698		344	
G3. Dispensed SSRI or SNRI and has a previous hospital admission for GI-bleeding or peptic ulcer disease (D)	195	30.8	212	36.3

- and the patient is also dispensed ≥1 of the following: antiplatelet, anticoagulant, NSAID (regardless of GI-protection) (N)	60		77	
**Total:** patients at risk of gastrointestinal bleeding (D)	10665	25.7	13668	17.0
**Total:** patient triggering the risk of gastrointestinal bleeding (N)	2746		2330	
**Bleeding**				
B1. Dispensed SSRI and has a previous hospital admission of a bleeding event (D)	165	39.4	218	32.1
- and the patient is also dispensed ≥1 of the following: anticoagulant or antiplatelet (N)	65		70	
B2. Dispensed SSRI and has a previous hospital admission for hemorrhagic stroke or for dementia (D)	192	51.0	139	36.0
- and the patient is also dispensed ≥1 of the following: anticoagulant or antiplatelet (N)	98		50	
**Total:** patients at risk of bleeding (D)	320	48.4	317	35.0
**Total:** patient triggering the risk of bleeding (N)	155		111	
**Constipation**				
CO1. Dispensed anticholinergic antidepressant TCA (dispensed strength ≥50 mg), maprotiline or paroxetine and aged ≥65 years (D)	2697	19.1	2316	21.3
- and the patient is also dispensed ≥2 further drugs known to have constipating effects (N)	514		494	
**Hyponatremia**				
H1. Dispensed SSRI or SNRI and aged ≥65 years (D)	10567	25.5	13551	22.2
- and the patient is also dispensed ≥2 further drugs known to cause hyponatremia (N)	2699		3014	
**Voiding disorders**				
VD1. Dispensed anticholinergic antidepressant TCA (dispensed strength ≥50 mg), maprotiline or paroxetine (D)	8125	3.0	6414	3.5
- and the patient is also dispensed ≥1 drug for benign prostatic hyperplasia or urinary retention (N)	241		226	
**Sleep disturbances/agitation**				
I. Dispensed SSRI or SNRI or MAOI or bupropion (D)	44814	17.8	60388	16.3
- and the patient is also dispensed ≥1 hypnotic (N)	7997		9842	
**Sexual dysfunction**				
SD1. Dispensed SSRI or SNRI (D)	44612	0.9	60252	1.2
- and the patient is also dispensed ≥1 drug for erectile dysfunction (N)	404		728	

Table S6: Proportion of antidepressant users triggering indicators targeting specific adverse drug reaction risks (2012) and their distribution among age groups

**Adverse drug reaction risks targeted by indicators**	No. of patients among each age-group triggering indicators				Total no. of patients (%)	Total no. of patients (%)	Total number of patients among all antidepressant users (**%**)
	18 - 39	40 - 64	65 - 79	80 - 100	18 - 64	65 - 100	
Falls/Fractures	0	0	8,078	3,997	0/51,144 (0.0)	12,075/22,456 (53.8)	12,075/73,600 **(16.4)**
Orthostatic hypotension/dizziness	0	0	4,843	2,255	0/51,144 (0.0)	7,098/22,456 (31,6)	7,098/73,600 **(9.6)**
Cardiovascular adverse effects	2,090	7,839	4,166	1,952	9,929/51,144 (19.4)	6,118/22,456 (27.2)	16,047/73,600 **(21.8)**
Hyponatremia	0	0	1,784	915	0/51,144 (0.0)	2,699/22,456 (12.0)	2,699/73,600 **(3.7)**
Insomnia	1,336	4,248	1,670	743	5,584/51,144 (10.9)	2,413/22,456 (10.7)	7,997/73,600 **(10.9)**
Any bleeding	2	33	1,868	929	35/51,144 (0.1)	2,797/22,456 (12.5)	2,832/73,600 **(3.8)**
Delirium	0	2	776	191	2/51,144 (0.0)	967/22,456 (4.3)	969/73,600 **(1.3)**
Constipation	0	0	429	85	0/51,144 (0.0)	514/22,456 (2.3)	514/73,600 **(0.7)**
Serotonin syndrome	397	1,189	312	80	1,586/51,144 (3.1)	392/22,456 (1.7)	1,978/73,600 **(2.7)**
Voiding disorders	1	71	130	39	72/51,144 (0.1)	169/22,456 (0.8)	241/73,600 **(0.3)**
Sexual dysfunction	22	322	55	5	344/51,144 (0.7)	60/22,456 (0.3)	404/73,600 **(0.5)**
Cognitive decline	0	15	41	33	15/51,144 (0.0)	74/22,456 (0.3)	89/73,600 **(0.1)**

Table S7: Proportion of antidepressant users triggering indicators targeting specific adverse drug reaction risks (2019) and their distribution among age groups

**Adverse drug reaction risks targeted by indicators**	No. of patients among each age-group triggering indicators				Total no. of patients (%)	Total no. of patients (%)	Total number of patients among all antidepressant users (**%**)
	18 - 39	40 - 64	65 - 79	80 - 100	18 - 64	65 - 100	
Falls/Fractures	0	0	10,069	4,754	0/65,324 (0.0)	14,823/27,277 (54.3)	14,823/92,601 **(16.0)**
Orthostatic hypotension/dizziness	0	0	5,510	2,439	0/65,324 (0.0)	7,949/27,277 (29.1)	7,949/92,601 **(8.6)**
Cardiovascular adverse effects	1,772	6,455	3,519	1,273	8,227/65,324 (12.6)	4,792/27,277 (17.6)	13,019/92,601 **(14.1)**
Hyponatremia	0	0	2,136	878	0/65,324 (0.0)	3,014/27,277 (11.0)	3,014/92,601 **(3.3)**
Insomnia	1,951	5,341	1,896	654	7,292/65,324 (11.2)	2,250/27,277 (9.3)	9,842/92,601 **(10.6)**
Any bleeding	3	48	1,558	786	51/65,324 (0.1)	2,344/27,277 (8.6)	2,395/92,601 **(2.6)**
Delirium	0	0	645	138	0/65,324 (0.0)	783/27,277 (2.9)	783/92,601 **(0.8)**
Constipation	0	0	414	80	0/65,324 (0.0)	494/27,277 (1.8)	494/92,601 **(0.5)**
Serotonin syndrome	487	1,633	387	85	2,120/65,324 (3.2)	472/27,277 (1.7)	2,592/92,601 **(2.8)**
Voiding disorders	0	59	133	34	59/65,324 (0.1)	167/27,277 (0.6)	226/92,601 **(0.2)**
Sexual dysfunction	81	512	130	5	593/65,324 (0.9)	135/27,277 (0.5)	728/92,601 **(0.8)**
Cognitive decline	0	11	24	34	11/65,324 (0.0)	58/27,277 (0.2)	69/92,601 **(0.1)**

Table S8: Potential deprescribing indications (PDIs) (i.e., simultaneous long-term and potential high-risk use) in 2012 and 2019

	**Second quarter 2012**	**Second quarter 2019**		
	No. of patients (crude %)	No. of patients (crude %, **age-sex standardised[a] %)**	**Relative risk 2019 vs 2012** (95% CI) Crude	Age-sex stand.
**PDI among AD users:**				
PDI	17,465/73,600 (23.7)	23,885/92,601 (25.8; **26.5)**	1.09 (1.07-1.11)	1.11 (1.10-1.13)
**Sex**				
Women	12,375/51,083 (24.2)	16,745/62,556 (26.8; **27.3)**	1.10 (1.08-1.13)	1.13 (1.10-1.15)
Men	5,090/22,517 (22.6)	7,140/30,045 (23.8; **24.6)**	1.05 (1.02-1.08)	1.09 (1.05-1.13)
**Age groups (years)**				
18 - 39	1,298/14,179 (9.2)	1,806/20,831 (8.7; **9.1)**	0.95 (0.88-1.01)	1.00 (0.93-1.07)
40 - 64	7,310/36,965 (19.8)	9,382/44,493 (21.1; **20.8)**	1.07 (1.04-1.10)	1.05 (1.02-1.08)
65 - 79	6,296/15,710 (40.1)	9,159/19,310 (47.4; **47.5)**	1.18 (1.16-1.21)	1.19 (1.16-1.22)
≥80	2,561/6,746 (38.0)	3,538/7,967 (44.4; **44.5)**	1.17 (1.12-1.22)	1.17 (1.13-1.22)
**High-risk use among each AD drug class**				
SSRI	10,819/39,791 (27.2)	15,165/51,244 (29.6; **30.7)**	1.09 (1.07-1.11)	1.13 (1.11-1.15)
TCA	6,086/25,198 (24.2)	7,066/25,833 (27.4; **27.4)**	1.13 (1.10-1.17)	1.14 (1.10-1.17)
SNRI	1,943/5,092 (38.2)	3,721/9,470 (39.3; **38.9)**	1.03 (0.99-1.07)	1.02 (0.97-1.07)
NASSA	1,805/6,865 (26.3)	3,816/13,279 (28.7; **28.1)**	1.09 (1.04-1.15)	1.07 (1.01-1.13)
MAOI	34/160 (21.3)	34/128 (26.6; **27.5)**	1.25 (0.83-1.89)	1.29 (0.88-1.91)
Others	649/2,586 (25.1)	812/2,700 (30.1; **27.8)**	1.20 (1.10-1.31)	1.11 (1.01-1.22)
**Deprivation quintile[b]**				
1 (most deprived)	3,896/15,599 (25.0)	5,360/20,109 (26.7; **27.6**)	1.07 (1.03-1.11)	1.10 (1.06-1.15)
2	3,737/15,120 (24.7)	4,984/19,099 (26.1; **26.8**)	1.06 (1.02-1.10)	1.09 (1.04-1.13)
3	3,289/13,594 (24.2)	4,343/16,890 (25.7; **26.2**)	1.06 (1.02-1.11)	1.08 (1.04-1.13)
4	3,759/16,065 (23.4)	5,042/19,081 (26.4; **27.0**)	1.13 (1.09-1.17)	1.15 (1.11-1.20)
5 (least deprived)	2,189/9,856 (22.2)	3,058/12,122 (25.2; **25.3**)	1.14 (1.08-1.19)	1.14 (1.08-1.20)
**Residence[b,c]**				
Large Urban area	4,227/16,470 (25.7)	5,467/20,278 (27.0; **27.7**)	1.05 (1.01-1.09)	1.08 (1.04-1.12)
Urban area	7,645/32,305 (23.7)	10,604/40,939 (25.9; **26.7**)	1.09 (1.07-1.12)	1.13 (1.10-1.16)
Accessible rural area	4,423/18,953 (23.3)	5,929/23,187 (25.6; **25.8**)	1.10 (1.06-1.13)	1.11 (1.07-1.15)
Remote rural area	575/2,506 (22.9)	787/2,897 (27.2; **27.9**)	1.18 (1.08-1.30)	1.21 (1.10-1.34)

[a]direct age-sex standardisation to the 2012 population, [b]deprivation and residence missing for 3,366 (4.6%) antidepressant users in 2012 and 5,300 (5.7%) in 2019, [c]Scottish Executive Urban-Rural Classification

Table S9: Sensitivity analysis for long-term and potential high-risk antidepressant use in 2012 and 2019

	**Second quarter 2012**	**Second quarter 2019**		
	No. of patients (crude %)	No. of patients (crude %, **age-sex standardised[a] %)**	**Relative risk 2019 vs 2012** (95% CI) Crude	Age-sex stand.
**Long-term AD use (SA1)**	28,134/73,600 (38.2)	45,165/92,601 (48.8; **49.8)**	1.28 (1.26-1.29)	1.30 (1.29-1.32)
Indicator F1:				
Co-prescribed ≥1 FRID	11,673/22,456 (52.0)	14,026/27,277 (51.4; **51.4**)	0.99 (0.97-1.01)	0.99 (0.97-1.01)
Co-prescribed ≥2 FRIDs	6,895/22,456 (30.7)	7,681/27,277 (28.2; **28.1**)	0.92 (0.89-0.94)	0.91 (0.89-0.94)
Co-prescribed ≥3 FRIDs	3,210/22,456 (14.3)	3,300/27,277 (12.1; **12.0**)	0.85 (0.81-0.89)	0.84 (0.80-0.88)
**Potential high-risk use (SA2)**	26,799/73,600 (36.4)	30,225/92,601 (32.6; **33.2**)	0.90 (0.88-0.91)	0.91 (0.90-0.92)
Indicator C5:				
Co-prescribed ≥1 drug with TdP risk	14,970/73,600 (20.3)	11,336/92,601 (12.2; **12.4**)	0.60 (0.59-0.62)	0.61 (0.60-0.63)
Co-prescribed ≥2 drug with TdP risk	6,774/73,600 (9.2)	4,533/92,601 (4.9; **5.0**)	0.53 (0.51-0.55)	0.54 (0.52-0.56)
Co-prescribed ≥3 drug with TdP risk	2,379/73,600 (3.2)	1,446/92,601 (1.6; **1.6**)	0.48 (0.45-0.52)	0.49 (0.46-0.53)
**Potential high-risk use (SA3)**	23,018/73,600 (31.3)	28,021/92,601 (30.3; **30.8**)	0.97 (0.95-0.98)	0.99 (0.97-1.00)
**Potential high-risk use (SA4)**	7,586/73,600 (10.3)	8,668/92,601 (9.4; **9.6**)	0.91 (0.88-0.94)	0.93 (0.90-0.96)
**Potential deprescribing indications (sPDI)**	4,223/73,600 (5.7)	5,990/92,601 (6.5; **6.7**)	1.13 (1.09-1.17)	1.16 (1.11-1.21)

[a]direct age-sex standardisation to the 2012 population; **SA1**: Use of antidepressants in each of 8 consecutive quarters (i.e., without grace periods); **SA2**: Proportion of patients triggering ≥1 high-risk use considering co-prescription of antidepressants with two or more fall risk increasing drugs (Indicator F1); **SA3:** Proportion of patients triggering ≥1 high-risk use considering co-prescription with two or more drugs known to increase the risk of torsades des pointes (Indicator C5); **SA4**: Proportion of patients triggering ≥1 high-risk use considering only indicators with the highest consensus ratings (median of 8 or 9 on a 9-point Likert scale) within the expert panel; **sPDI**: Proportion of patients triggering long-term use without grace periods AND triggering ≥1 high-risk use considering only indicators with the highest consensus ratings.
